# A critical review of the pharmacological treatment of REM sleep behavior disorder in adults: time for more and larger randomized placebo-controlled trials

**DOI:** 10.1007/s00415-020-10353-0

**Published:** 2021-01-07

**Authors:** Moran Gilat, Nathaniel S. Marshall, Dries Testelmans, Bertien Buyse, Simon J. G. Lewis

**Affiliations:** 1 Neurorehabilitation Research Group (eNRGy), Department of Rehabilitation Sciences, KU Leuven, Tervuursevest 101, box 1501, 3001 Leuven, Belgium; 2 Woolcock Institute of Medical Research, University of Sydney, Sydney, Australia; 3 Centre for Sleep and Wake Disorders (LUCS), Department of Pneumology, Leuven University, UZ Leuven, Leuven, Belgium; 4 Forefront Parkinson’s Disease Research Clinic, Brain and Mind Centre, School of Medical Sciences, University of Sydney, Sydney, Australia

**Keywords:** Pharmacotherapy, Drugs, Parkinson’s disease, Lewy body dementia, Multiple system atrophy, Benzodiazepines, Circadin

## Abstract

Rapid Eye Movement sleep behavior disorder (RBD) is a parasomnia causing sufferers to physically act out their dreams. These behaviors can disrupt sleep and sometimes lead to injuries in patients and their bed-partners. Clonazepam and melatonin are the first-line pharmacological treatment options for RBD based on direct uncontrolled clinical observations and very limited double-blind placebo-controlled trials. Given the risk for adverse outcomes, especially in older adults, it is of great importance to assess the existing level of evidence for the use of these treatments. In this update, we therefore critically review the clinical and scientific evidence on the pharmacological management of RBD in people aged over 50. We focus on the first-line treatments, and provide an overview of all other alternative pharmacological agents trialed for RBD we could locate as supplementary materials. By amalgamating all clinical observations, our update shows that 66.7% of 1,026 RBD patients reported improvements from clonazepam and 32.9% of 137 RBD patients reported improvements from melatonin treatment on various outcome measures in published accounts. Recently, however, three relatively small randomized placebo-controlled trials did not find these agents to be superior to placebo. Given clonazepam and melatonin are clinically assumed to majorly modify or eliminate RBD in nearly all patients—there is an urgent need to test whether this magnitude of treatment effect remains intact in larger placebo-controlled trials.

**Supplementary Information:**

The online version of this article (10.1007/s00415-020-10353-0) contains supplementary material, which is available to authorized users.

## Introduction

Rapid Eye Movement (REM) sleep behavior disorder (RBD) is a parasomnia, in which a loss of physiological muscle atonia during REM sleep leads to dream enactment behaviors (DEB) [1]. A clinical history of DEB together with video-polysomnography (PSG) confirmed REM sleep without atonia (RWA), or a combination of RWA and dream-enactment behaviors documented with PSG, are mandatory for a clinical diagnosis of RBD according to the International Classification of Sleep Disorders-III. Although RBD symptoms can be seen in several disorders, such as narcolepsy-cataplexy and parasomnia overlap disorder, and may be precipitated by certain drugs, such as selective serotonin reuptake inhibitors [1], its isolated presence in the general adult population is closely linked with alpha synuclein neuropathology and a future diagnosis of either Parkinson’s Disease (PD), Dementia with Lewy Bodies (DLB) or Multiple System Atrophy (MSA) [2, 3]. In the general population around 1% of people have clinically isolated RBD, whereas the proportion is much higher in PD (20–50%) and over 80% of DLB and MSA patients report RBD [1, 3]. People with severe obstructive sleep apnea (OSA) frequently experience OSA-induced arousals that may result in movements during REM sleep mimicking RBD symptoms. Importantly, however, people with OSA can indeed have true RBD, which should be confirmed with PSG after treating the OSA [4].

Although patients may not be aware of mild symptoms [5], treating RBD can often be necessary as it can cause frequent and sometimes life-threatening injuries to patients and their bed partners [1, 6]. The current guidelines for RBD treatment include counseling, modification of the bedroom environment to reduce the risk of injury and two main pharmacological agents, namely clonazepam and melatonin [1, 7]. Such information is also presented to patients via reputable online sources, such as sleepfoundation.org and websites of specialized sleep clinics.

Clonazepam has been the recommended treatment since the first clinical description of RBD by Schenck and colleagues back in 1986 [8]. Indeed, subsequent case series and open-label studies have reported a clinical efficacy rate of up to 90% [9]. Clonazepam is a benzodiazepine, which enhances inhibitory γ-aminobutyric acid (GABA) activity in the central nervous system leading to anticonvulsant, anxiolytic and skeletal muscle relaxation effects. It has been suggested that clonazepam may be efficacious by suppressing phasic bursts of muscle activity during REM sleep [10]. However, the true mechanisms of action of clonazepam for reducing RBD remain unknown [11, 12]. Significantly, clonazepam is a long acting benzodiazepine with a half-life of 30–40 h that should be used with caution, especially in older adults, as it can lead to dependence along with frequent and sometimes serious side effects, including confusion, morning sedation, cognitive impairment and falls [7]. Clonazepam may also induce [13] or possibly worsen obstructive sleep apnoea (OSA) symptoms [1, 7]. It is, therefore, critical to systematically assess whether the existing evidence supports the use of clonazepam to treat RBD, especially in the older population who are known to have the highest prevalence of RBD and in whom the adverse outcomes, such as falls, may be most impactful, particularly in patients with a neurodegenerative disorder such as PD or DLB [5].

When compared to clonazepam, melatonin has a much safer profile with no reports of dependence, along with fewer and milder side effects, which include headache and morning sleepiness [1, 7]. Melatonin has, therefore, been proposed as a preferable treatment for RBD, especially for older patients and/or those who have OSA, neurodegenerative conditions, are at higher risk of experiencing side effects, or are considered refractory to the effects of clonazepam [1, 7, 14–18].

Melatonin is a natural hormone that is predominantly synthesized in the pineal gland and promotes sleep propensity across the brain [19]. Endogenous melatonin secretion is tightly regulated by photic cues received by the hypothalamic suprachiasmatic nucleus, which is the major circadian oscillator [19]. Melatonin secretion reduces with ageing and thus, low dosages (0.3–1 mg) of exogenous melatonin may help to coordinate circadian rhythms when administered in a specifically timed manner [19, 20]. This may be of particular interest to people with PD, who experience circadian dysregulation [21] and have altered peak melatonin concentration levels [22, 23]. The soporific effect of acutely administering higher dosages (2-25 mg) of melatonin at night-time has further been shown to improve sleep efficiency and may help reduce secondary sleep disorders, including RBD, whereby its chronobiotic effect may correct the timing, amount, and quality of REM sleep when administration is timed correctly [19, 20]. There is also indication of melatonin reducing the amount of RWA in patients with RBD [20]. Melatonin has a short (30–50 min) elimination half-life, which lessens ‘hang-over’ effects the following morning [19]. Due to its short half-life, however, the effectiveness of melatonin might be suboptimal for the majority of REM sleep periods that occur in the second part of the night. A prolonged release formulation that releases melatonin gradually over 8–10 h (Neurim Pharmaceuticals Inc.: Circadin) has, therefore, been proposed for treating RBD [17].

Despite the widespread use of both clonazepam and melatonin for treating RBD, until recently there was a lack of good quality trial data [24]. Current international guidelines are still based on evidence from mainly small case-series and open-label studies [25], which are at high risk of bias and have demonstrated inconsistencies in the clinical effectiveness levels reported. In fact, many patients with RBD were considered refractory to these first-line treatment options, which led clinicians to trial a multitude of other pharmacological agents off-label [26–29]. Past reviews have not consistently accounted for such reports when calculating the number of responders for these commonly prescribed treatments [25, 30], or were conducted more than 10 years ago [7]. Thus, there is a need to re-assess whether the existing evidence supports the current recommended guidelines for the pharmacological management of RBD.

The purpose of this update is to provide a semi-systematic overview of all clinical and scientific evidence published to date on the pharmacological management of RBD. We focus on the middle- to older (50 years and above) adult population, which has the highest prevalence of RBD as well as being at the greatest risk for adverse outcomes [5]. Doing so, we set out to provide an update on the total number of adults with isolated or secondary RBD who were clinically followed to assess the efficacy of any pharmacological compound given to treat their RBD symptoms. We also assessed the level of evidence based on the study design, as depicted in *Box 1*. This review specifically investigates the divide between clinical expectancy and the actual evidence for the effectiveness of the common drugs being recommended for managing RBD. We also seek to provide future directions on how this field could move to a more rigorous evidence-base.
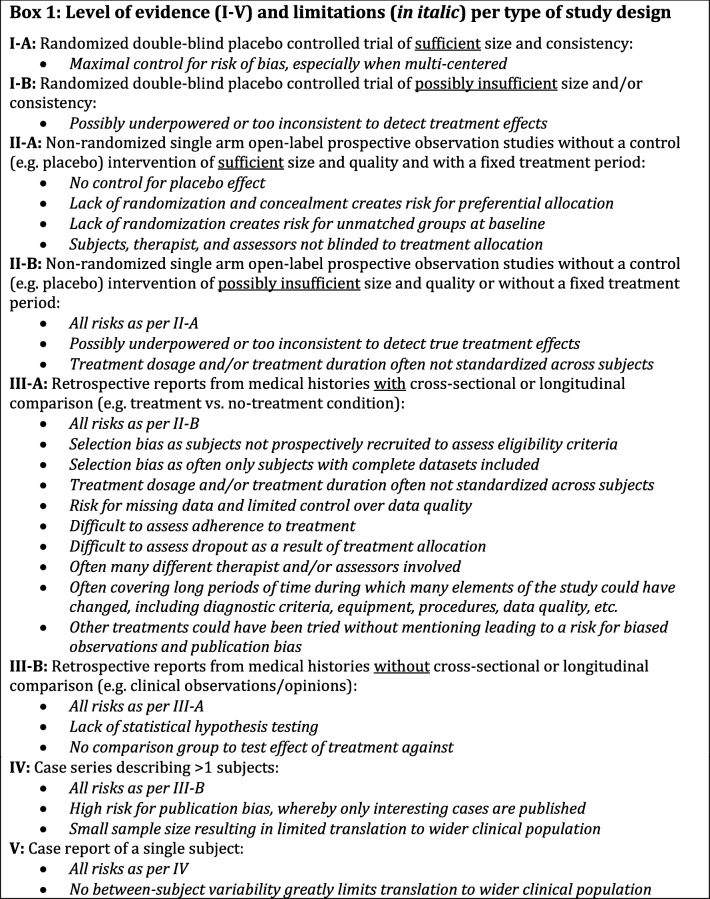


## Search syntax and screening

**Fig. 1:**
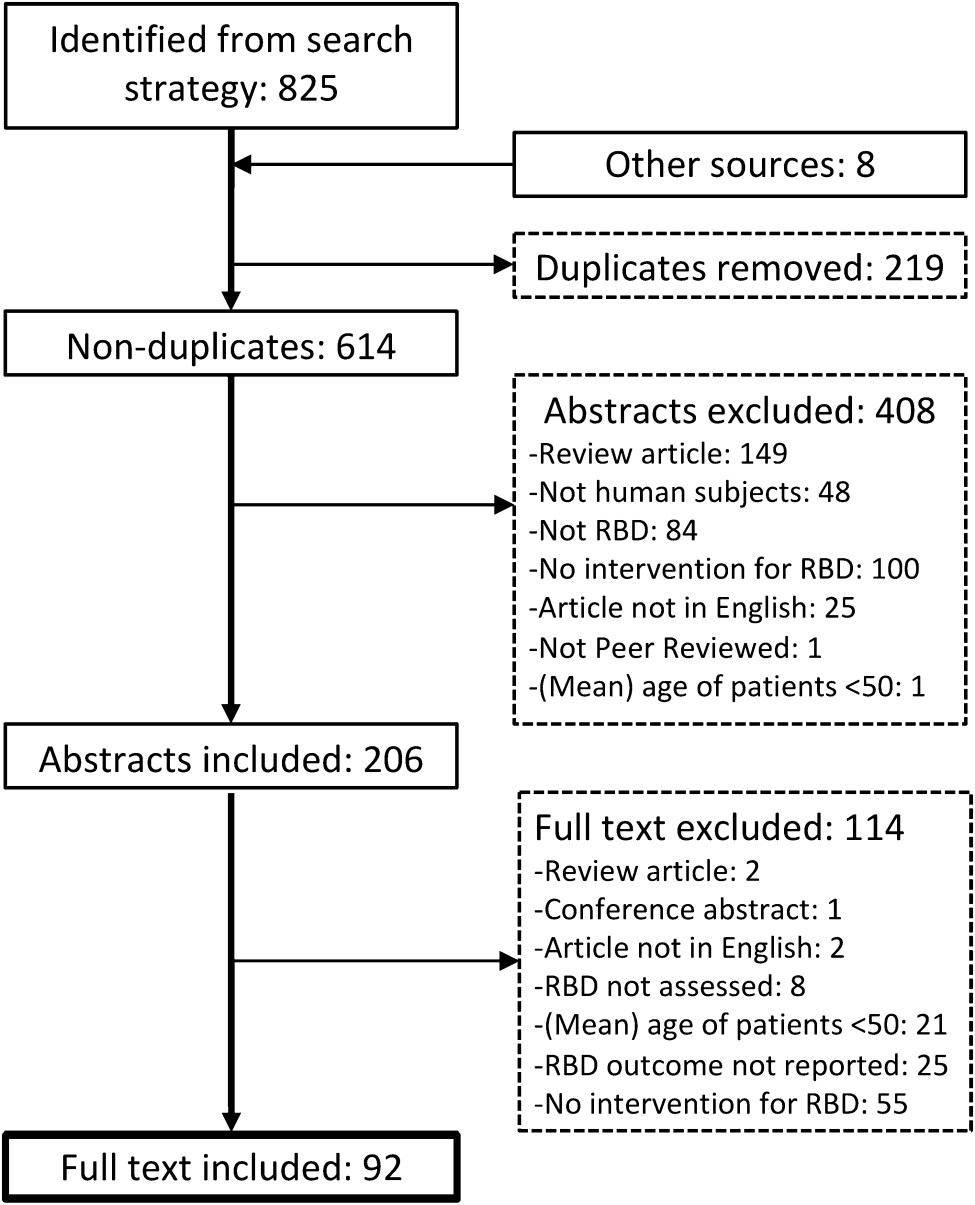
Flowchart of search results and screening

Literature was searched in PubMed, EMBASE, MEDLINE Ovid, and Web of Science core collection from conception until the 17th April 2020. The following terms were used to search in all fields, namely: ((REM sleep behavior disorder OR REM behavior disorder OR RBD); AND (medication OR drug OR treatment OR therapy OR pharmacotherapy OR pharmacological OR intervention); AND (clonazepam OR melatonin OR temazepam OR lorazepam OR zolpidem OR zopiclone OR pramipexole OR donepezil OR ramelteon OR agomelatine OR cannabinoid OR sodium oxybate OR dopamine agonist OR levodopa)) (Fig. 1).

A total of 607 non-duplicate citations were identified by the search strategy and an additional eight citations, of which five eligible [31–35], were identified from the reference list of a previous review on the best practice guides for RBD [7]. Two reviewers (MG, DT) then screened the abstracts and remaining full-texts according to the following inclusion criteria: (i) Any type of study containing original data on a change in RBD symptom severity following any type of pharmacological intervention administered with the purpose of reducing RBD in any type of disorder or isolated RBD; (ii) Intervention administered for more than 1 week; (iii) RBD severity assessed as an outcome of the intervention, including surrogate measures, such as RWA, and clinical opinions; (iv) The mean or median age of the RBD group investigated was > 50 years, or the age of the persons with RBD in case report series were > 50 years at the time of the assessment; (v) Written in English language; (vi) Published in a peer-reviewed scientific journal, and; (vii) Evidence based on human subjects. The following exclusion criteria were additionally applied to assess final eligibility of remaining full-texts: (i) Review of the literature with or without meta-analysis; (ii) Conference abstract; (iii) Intervention outcome on RBD not reported.

## Risk of bias assessment

To aid interpretation of intervention outcomes, the study quality assessment tool for controlled intervention studies by the NIH, National Heart, Lung, and Blood Institute (nhlbi.nih.gov) was used to assess risk of bias for each of the RCTs performed. This tool assesses 14 criteria to help evaluate internal validity and detect possible flaws in study design. The risk of bias assessment was conducted by MG and controlled for accuracy by DT.

## Publication bias assessment

To assess for possible publication bias, another search was conducted on 20 November 2020 in the International Clinical Trials Registry Platform of the World Health Organisation (apps.who.com), which encompasses many of the trial registries around the world, including ClinicalTrials.gov. We used the search terms (‘REM sleep behavior disorder’ OR ‘RBD’), which led to a total of 77 listed trials that were screened according to the same inclusion criteria as described above, except criteria v and vi. A total of 17 trials were deemed eligible and assessed for possible publication bias.

## Literature search results

A total of 92 articles were deemed eligible for inclusion in the review (Fig. 1).

## Study designs

**Fig. 2:**
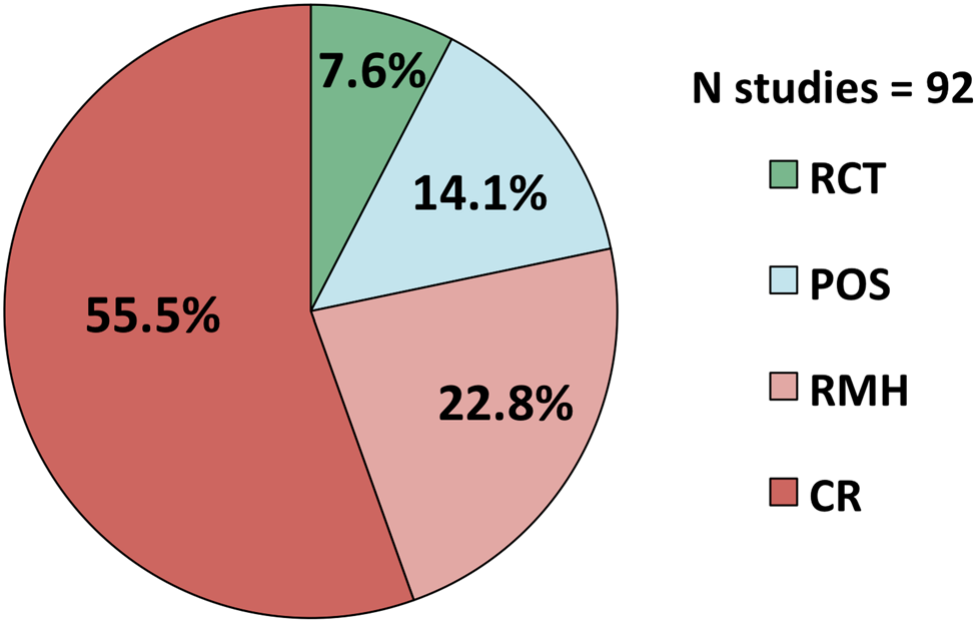
Overview of study designs used to test pharmacological interventions for treating RBD in the adult population. Abbreviations: RCT = Randomized controlled trial; POS = Prospective single arm open-label cohort study; RMH = Retrospective study based on medical history; CR = Case report

As shown in Fig. 2, the large majority of included studies were case reports (CR, *n* = 51) or retrospective accounts based on medical histories (RMH, *n* = 21). Only 7 studies had a single-centered randomized placebo-controlled trial (RCT) design and 13 were prospectively planned single arm open-label (i.e., without a control intervention) cohort studies (POS). Overall, the bulk of evidence that currently exists on the pharmacological management of RBD in the adult population is, therefore, considered to be of poor scientific quality (*Box 1*).

## Overview of results

The included papers were divided into supposedly prospectively planned studies (i.e., noted by the author as being prospective, but not necessarily pre-registered; *n* = 20) and retrospective studies or case reports (*n* = 72). Table 1 presents the full systematic overview of the prospective studies per drug class and type including the level of evidence as per *Box 1*. Table 2 presents an overview of the risk of bias for each of the RCTs. Given the intrinsically high risk of bias for POS, and especially RMH and CR (see *Box 1*), no quality assessment was conducted on those studies.

**Table 1:** Systematic overview of all prospective observational studies on the pharmacological management of RBD

Study Design	Study (First author, year)		Clinical population	Treatment (Daily dosage)	Groups (n subjects) [Age in years]	RBD screening method	RBD outcome measure(s)	Main results on RBD	Level of evidence (*Box 1*)
Clonazepam									
DB-RCT	Shin, 2019 [36]		Parkinson	(A) Clonazepam (0.5 mg) daily for 4 weeks (B) Matched placebo (0.5 mg) daily for 4 weeks	(A) Active (n = 19) [66, 48–73]* (B) Placebo (n = 20) [70, 56–77]	RBD single screening question	(1) CGI	Non-effective No other outcomes of RBD assessed	I-B
POS	Li, 2016 [37]		iRBD	Clonazepam (0.125–1.0 mg) at baseline and (0.125–3.0 mg) at follow up 28.8 ± 13.3 months later *Treatment duration not fixed*	Active (n = 39) [68.3 ± 7.8] -27 remained on therapy -10 had prescription changed -2 were not on therapy at follow-up *No control group*	PSG	(1) RWA on PSG (2) Movements during sleep on PSG (3) RBDQ-HK modified to cover past 3 months	Worse RWA and no reduction in movements during sleep on PSG. Subjective improvement reported in 66.7% of subjects	II-B
POS	Iranzo, 2005 [39]		iRBD, Parkinson, and MSA	If clinically required, clonazepam (0.25–0.5 mg) was started at diagnosis and titrated up in 0.25–0.5 mg increments to clinical response and tolerability. Mean follow-up across all subjects was 26.9 ± 21.3 months later *Treatment duration not fixed*	Active -iRBD (n = 39) taking mean dose of 0.89 (0.55) mg/day [68.4 ± 5.9] -Parkinson (n = 45) taking mean dose of 0.72 (0.42) mg/day [64.8 ± 7.8] -MSA (n = 26) taking mean dose of 0.63 (0.22) mg/day [62.0 ± 7.1] *No control group*	PSG	(1) Clinical response to clonazepam (Substantial, partial, no response, in %)	Subjective improvement (substantial or partial) reported in almost all subjects, except 3.1% of the iRBD cases. No side effects in Parkinson, whereas 11.1% of MSA and 25% of iRBD reported side effects, mainly somnolence	II-B
POS	Lappiere, 1992 [38]		iRBD, with one case presenting soft cerebellar signs on MRI	Clonazepam (0.5–2.0 mg) for a duration of 2 months	-Active (n = 5) [58.6, 44–65]* *No control group*	PSG	(1) RBD episodes on PSG (2) Phasic EMG density on PSG at REM (3) RSWA on PSG	(1) Subjective improvements reported though occasional sleep-talking and limb-jerking observed (2) Reduction in phasic EMG density at REM (3) No reduction in RSWA	II-B
Melatonin									
DB-RCT	Gilat, 2020 [42]		Parkinson	(A) PR-melatonin (Circadin, 2 × 2 mg) for 8 weeks (B) Matched placebo (2 × 2 mg) for 8 weeks	(A) Active (n = 15) [65.3 ± 6.9] (B) Placebo (n = 15) [67.9 ± 5.3]	PSG	(1) Frequency of RBD in 2nd month of treatment based on self-report diary entries (2) RWA on PSG in a subset of n = 8 on melatonin and n = 6 on placebo (3) Several RBD questionnaires (4) CGI-I	Non-effective Also no group differences seen for RWA on PSG, the CGI, or any of the RBD Questionnaires	I-B
DB-RCT	Jun, 2019 [41]		i-RBD	(A) PR-melatonin (Circadin, 1 × 2 mg) + matched placebo (2 × 2 mg) for 4 weeks (B) PR-melatonin (Circadin, 3 × 2 mg) for 4 weeks (C) Matched placebo (3 × 2 mg) for 4 weeks	(A) Active 1 (n = 7) [68.1 ± 9.1] (B) Active 2 (n = 9) [64.7 ± 8.3] (C) Placebo (n = 9) [66.4 ± 8.5]	PSG	(1) CGI-I (2) RBDQ-KR questionnaire after 4 weeks of treatment (3) DEB frequency recorded by patients on daily diary	Non-effective on either primary- or any secondary outcomes on RBD, including diary entries. RWA on PSG not assessed post-treatment	I-B
DB-RCT	Kunz, 2010 [16]		Mixed diagnoses (5 iRBD, 1 Parkinson, 2 Narcolepsy + PLMS)	(A) Melatonin (3 mg) for 4 weeks (B) Placebo for 4 weeks	Subjects (n = 8) entered in cross-over study and randomized to first receive melatonin or placebo therapy for 4 weeks and switch treatments after 3–5 days of washout period [53.8, 26–67]* *Two subjects were 26 and 37 years old, respectively*	PSG	(1) Number of REM epochs without RWA on PSG (2) CGI severity	Compared to baseline, melatonin significantly reduced number of REM epochs with RWA and improved CGI, whereas the improvement seen during placebo did not reach significance. No differences were found for RWA or CGI severity scores when comparing melatonin to placebo	I-B
POS	Takeuchi, 2001 [46]		RBD, idiopathic or with unknown concomitant diagnoses (not reported)	Melatonin (3 mg) that in some subjects was titrated up to 9 mg according to degree of clinical RBD symptoms *Treatment duration not fixed and not reported*	Total of 15 subjects with RBD assessed at baseline and at a non-specified point in time during therapy ‘when their clinical symptoms were improved or stable’ [63.5, SD or range not reported]	PSG	(1) Clinical opinion (2) % Tonic/Phasic REM activity on PSG (3) Melatonin blood concentration levels at 3hour intervals	Remarkable improvement noted in 3/15 and partial improvement in 10/15 patients. Significant reduction in tonic REM EMG activity on PSG. Melatonin concentration increased in a subset of patients with low baseline melatonin levels	II-B
POS	Kunz, 1999 [15]		Mixed diagnoses (2PD, 2 iRBD, 1 RBD with hypertension, 1 RBD with sympathetic dysautonomia)	Melatonin (3 mg) for 6 weeks	Total of 6 subjects assessed before and after therapy [54, 26–71]*	PSG	(1) Clinical opinion (2) Number of REM epochs without RWA on PSG (3) Movement time in bed based on actigraphy data	Subjective improvements in 5/6 patients with presumed long-term effects lasting weeks or even up to 22 months in one subject. Reduced REM epochs without muscle atonia on PSG seen after 6 weeks of melatonin compared to baseline	II-B
Ramelteon									
POS	Esaki, 2016 [48]		iRBD	Ramelteon (8 mg) for 8.3 ± 6.8 weeks *Treatment duration not fixed*	Active (n = 12) [70.9, 52–81] *No control group*	PSG	(A) RWA on PSG (B) RBDSS on PSG (C) VAS-scale for subjective RBD severity rated by partner	Non-effective on PSG or VAS-scale, though subjective severity trended towards a significant improvement. Some subjects with worsening RWA reported subjective improvements	II-B
POS	Kashihara, 2016 [49]		Parkinson	Ramelteon (8 mg) for 12 weeks	Active (n = 35) -24 screened positive for probable RBD -6 stopped therapy due to adverse events -3 were lost to follow-up [69.1 ± 11.1] *No control group*	RBDSQ (Japanese version)	RBDSQ (Japanese version)	Significant improvement in 13 patients with probable RBD, but also in 11 patients without probable RBD	II-B
Dopamine-agonists									
POS	Sasai, 2012 [51]		iRBD with PLMS	Pramipexole (0.21 ± 0.09 mg) for 9.1 ± 7.1 months *Treatment duration not fixed*	Total of 15 subjects assessed before and after treatment period [66.5, 57–75]	PSG	(1) Four-point severity scale based on clinical opinion (2) Subjective frequency of nightmares (3) RSWA on PSG	Subjective partial improvements were noted for 12/15 patients	II-B
POS	Kumru, 2008 [53]		Parkinson	Pramipexole (0.54 mg) divided in 3 dosages with last dosage taken one hour before bedtime, for 3 months	Total of 11 PD with untreated RBD on levodopa monotherapy at study entry assessed before and after 3 months of pramipexole therapy [62.1 ± 8.0]	PSG	(1) Three point severity scale on subjective frequency of RBD by patient and bed-partner (2) Subjective frequency of unpleasant dreams by patients and bed-partners (3) RWA on PSG 4) % of time spent with DEB during REM sleep (4) Three point severity scale of DEB on PSG by blinded assessors	Non-effective on both subjective and objective PSG measures	II-B
POS	Fantini, 2003 [54]		iRBD	Pramipexole (0.125 mg/24 hr) titrated up by 0.125 mg every 3 days until a mean final dosage of 0.78 ± 25 mg/24 hr, 1–9 months later *Treatment duration not fixed*	Total of 8 subjects with iRBD assessed before and after 4.5 (range 1–9.5) months of therapy [66 ± 6.8]	PSG	(1) Four point subjective severity rating based on patient and bed partner self-report of RBD severity on (2) RWA on PSG (3) DEB on PSG	Subjective sustained improvement in 5/8 patients and reduced simple DEB on PSG, though RWA on PSG worsened on therapy compared to baseline	II-B
POS	Wang, 2016 [50]		Parkinson	Rotigotine (2 mg/24 hr) titrated up to 16 mg over 8 weeks followed by 12–20 weeks of dose-maintenance *Treatment duration not fixed*	Active (n = 11) [66.27 ± 8.47]	PSG	(A) RWA on PSG (B) DEB on PSG (C) RBDQ-HK	Non-effective on PSG measures. Subjective improvement reported in 63.64% of subjects	II-B
POS	Dušek, 2010 [52]		Parkinson	PR-ropinirole for 5–13 weeks at the dosage closest to the dosage of immediate release ropinorole already taken by the subjects at study entry for past 3.4 ± 1 years *Treatment duration not fixed*	Total of 35 PD, of whom only 5 had RBD, taking immediate release ropinorole 2–5 times daily at study entry who were switched to a similar dose (17.2 ± 6 mg) of PR-ropinorole and followed-up 5–13 weeks later [62.5, 44–75]*	PSG	RBDSQ	Non-effective in subset of 5 PD with RBD at study entry	II-B
Acetylcholinesterase inhibitors									
SB-RCT	Brunetti, 2014 [28]		iRBD with MCI	(A) Rivastigmine patch (4.6 mg/24 hr) for 30 days (B) Placebo patch for 30 days	Subjects (n = 25) deemed refractory to melatonin or clonazepam therapy entered in cross-over study and randomized to first receive rivastigmine or placebo therapy for 30 days and switch treatments after 7 days of washout period [63.0, 49–81]*	PSG	(A) RBD frequency recorded on diary by bed-partners *PSG not performed post-therapy*	Improvement in subjective RBD frequency on rivastigmine compared to placebo	I-B
DB-RCT	Di Giacopo, 2012 [27]		Parkinson	(A) Rivastigmine patch (4.6 mg/24 hr) for 3 weeks (B) Placebo patch for 3 weeks	Subjects (n = 12) deemed refractory to melatonin or clonazepam therapy entered in cross-over study and randomized to first receive rivastigmine or placebo therapy for 3 weeks and switch treatments after 7 days of washout period. Two dropped out [66.7 ± 7.3]	PSG	(A) RBD frequency recorded on diary by bed-partners (B) RWA on PSG in subset of 4 subjects	Significantly lower frequency of RBD on diary during rivastigmine, but not placebo. No change on PSG in subset of 4 subjects	I-B
NMDA antagonist									
DB-RCT	Larsson, 2010 [47]		Parkinson with dementia (PDD) or DLB	(A) Memantine (5 mg) for 24 weeks titrated up to 20 mg at week 4 of therapy (B) Placebo *Secondary analysis from previously published DB-RCT, which was not focused on RBD*	(A) Active (n = 25) [76.4 ± 6.5] (B) Placebo (n = 22) [76.3 ± 5.0]	Probable RBD based on subjective rating on a single item of the Stavanger Sleep Questionnaire (SSQ): “Is the patient physically active during sleep?”	Four-point subjective severity rating on a single item of the SSQ regarding physical activity during sleep, which may or may not have occurred during REM sleep. The baseline frequency of patients with probably RBD was 54% and equally distributed between the two groups	Probable RBD severity decreased significantly following memantine compared to placebo, though careful interpretation is warranted due to possible non-RBD specificity of outcome measure	I-B
SSRI									
POS		Yamamoto, 2006 [33]	IRBD	Paroxetine (10-40 mg) for an unknown duration *Treatment duration and dosage not fixed*	(A) Active (n = 19) [64.7 ± 7.8] *No control group*	PSG	(A) Subjective RBD severity rating (Mild, Moderate, Severe)	Subjective RBD improved to a mild state in 11 and to a moderate state in 5 patients, while in 3 patients severe RBD persisted. Treatment was ceased in 2 due to side-effects	III-B

Daily dose presented as mean ± SD mg/day; Age in years presented as [mean ± SD], or [mean, range]; * = one or more subjects could be < 50 years of age

CGI = Clinical Global Impression scale; DB-RCT = Double-blinded randomized controlled trial; DEB = Dream Enactment Behaviors; (I)RBD = (Idiopathic) REM sleep Behavior Disorder; Parkinson = Parkinson’s disease; PLMS = Periodic Limb Movements; POS = Prospective open-label study (i.e., no control intervention); PR = Prolonged Release; PSG = Polysomnography; QA = Quality Assessment; RBDQ-HK = RBD Questionnaire Hong Kong; RBDSQ = RBD Screening Questionnaire; RBDSS = RBD Severity Scale based on PSG; RWA = REM sleep without atonia; SB-RCT = Single-blinded RCT; SSRI = Selective Serotonin Reuptake Inhibitor; VAS = Visual Analogue Scale

**Table 2:**
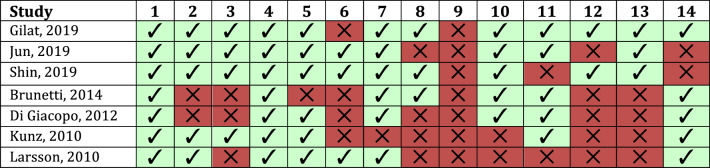
Quality assessment of the randomized controlled trials assessing the pharmacological management of RBD in adults

Summary of items from the NIH quality assessment tool (nhlbi.nih.gov): 1 = Randomized; 2 = Randomization adequate; 3 = Concealed; 4 = Blinding subjects; 5 = Blinding assessors; 6 = Groups matched at baseline; 7 = Overall dropout ≤ 20%; 8 = Differential dropout ≤ 15% between groups; 9 = Adherence to intervention; 10 = Other interventions avoided; 11 = Outcome assessed using valid and reliable measures; 12 = Sample size large enough for 80% power; 13 = outcomes and analyses pre-specified (registered); 14 = All received treatment allocated to. Green background with ‘✓’ = low risk of bias; Red background with ‘✕’ = high or unclear risk of bias

Table 3 presents a summary report of the updated total number of responders per pharmacological intervention trialed for treating RBD. Patients were considered full responders if the authors reported clear and sustained improvements for the duration of the trial without troublesome side effects or classified the patients as full responders, often because of > 50% symptom reduction (Clinical Global Impression-Improvement (CGI-I) score = (very) much improved); as partial responders if the authors reported improvements, but with some RBD symptoms remaining or some minimally troublesome side-effects occurring (CGI-I score = minimally improved); and as non-responders if the authors reported no sustained improvements for the duration of the trial, classified the patients as non-responders, or the treatment had to be discontinued due to troublesome side-effects (CGI-I score = no change, or worse). An overview of the data underlying Table 3 listing all clinical interpretations on the efficacy for each drug and dosage used to treat RBD in the adult population per study is presented in Supplementary Tables S1–S28. A description of the existing evidence for clonazepam and melatonin as the current first-line treatment options is provided below, and a description of the evidence for all other alternative drugs trialed to treat RBD off-label is provided in the Supplementary materials.

**Table 3:** Update on the total number of responders per drug used to treat RBD as based on clinical interpretation

Drug class	Drug type	*N* studies	*N* patients	YES *N* (%)	PARTIAL *N* (%)	NO *N* (%)
Benzodiazepine	Clonazepam^1^	51	1026	684 (66.7)	159 (15.5)	183 (17.8)
	Clonazepam + Melatonin	6	13	3 (23.1)	6 (46.1)	4 (30.8)
	Clonazepam + Adjunctive	10	69	41 (59.4)	3 (4.4)	25 (36.2)
	Temazepam	3	3	1 (33.3)	0 (0)	2 (66.7)
	Zopiclone	4	12	7 (58.3)	0 (0)	5 (41.7)
	Other	6	22	3 (13.6)	0 (0)	19 (86.4)
Melatonin (+ agonist)	Melatonin^1^	22	137	45 (32.9)	37 (27.0)	55 (40.1)
	Melatonin + Adjunctive	2	3	0 (0)	2 (66.7)	1 (33.3)
	Ramelteon	3	16	5 (31.3)	1 (6.2)	10 (62.5)
	Agomelatine	1	3	3 (100)	0 (0)	0 (0)
Dopamine (+ agonist)	Levodopa	4	45	8 (17.8)	1 (2.2)	36 (80)
	Pramipexole	6	126	71 (56.3)	4 (3.2)	51 (40.5)
	Ropinirole	4	7	0 (0)	1 (14.3)	6 (85.7)
	Rotigotine	1	11	7 (63.6)	0 (0)	4 (36.4)
Anticholinergic	Donepezil	4	56	1 (1.8)	3 (5.4)	52 (92.8)
	Rivastigmine	3	36	25 (69.4)	1 (2.8)	10 (27.8)
NMDA antagonist	Memantine	1	24	NR	NR	NR
Gabapentinoid	Gabapentine	3	16	12 (75)	0 (0)	4 (25)
	Pregabalin	1	3	2 (66.7)	0 (0)	1 (33.3)
Noradrenergic agonist	Clonidine	2	2	1 (50)	0 (0)	1 (50)
Antidepressants (per class)	SSRI	5	24	0 (0)	17 (70.8)	7 (29.2)
	Tricyclic	6	9	1 (11.1)	0 (0)	8 (88.9)
	Other	3	8	0 (0)	0 (0)	8 (100)
Antipsychotics	Mixed types	6	9	3 (33.3)	1 (11.1)	5 (55.6)*
Anticonvulsants	Phenobarbital	1	1	0 (0)	0 (0)	1 (100)
	Lamotrigine	1	1	0 (0)	0 (0)	1 (100)
	Oxcarbazepine	1	1	0 (0)	0 (0)	1 (100)
Gamma-hydroxybutyric acid	Sodium oxybate	4	4	4 (100)	0 (0)	0 (0)
	Sodium oxybate + Pramipexole	1	1	1 (100)	0 (0)	0 (0)
Other	Yi-Gan San	2	18	13 (72.2)	0 (0)	5 (27.8)
	Yi-Gan San + Adjunctive	1	19	4 (21.1)	0 (0)	15 (78.9)
	Cannabidiol	1	4	4 (100)	0 (0)	0 (0)
	Aspirin	1	1	0 (0)	0 (0)	1 (100)
	Metropolol	1	1	0 (0)	0 (0)	1 (100)

% = Percentage of total sample per drug type; YES = Full responders, authors reported clear and sustained improvements without side effects; PARTIAL = Partial responders, authors reported improvements, but with some RBD symptoms remaining or some non-troublesome side-effects occurring; NO = Non-responders, authors reported no sustained improvement or the treatment was discontinued due to troublesome side-effects; 1 = Currently the first-line treatment options; * = Some of the antipsychotic drugs induced or worsened RBD. Abbreviations: SSRI = Selective Serotonin Reuptake Inhibitor; NMDA = *N*-Methyl-D-aspartate; NR = Not reported

## First-line treatment options

### Clonazepam

Amalgamating all clinical accounts suggested that 684 (66.7%) out of a total of 1026 RBD patients (regardless of aetiology) reported clear benefits following clonazepam mono-therapy with no troublesome side effects noted by the authors for the duration of the trial. A further 159 (15.5%) reported partial improvements with some residual RBD symptoms or manageable side effects occurring, while a total of 183 (17.8%) of RBD patients were considered refractory, experiencing intolerable side effects or showing no sustained reduction of their RBD for the duration of the trial. This update is based on the clinical reports from 1 RCT [36], 3 prospective observational studies (POS), 16 retrospective medical histories (RMH), and 31 case reports (CR). The patients across these studies represented a mixture of iRBD and secondary RBD with comorbid conditions (Table S1).

When clonazepam mono-therapy proved ineffective, clinicians often resolved to trial in their case series a combination of clonazepam with one or multiple other pharmacological agents, as the second treatment option. A total of 13 RBD patients were reported to receive a combination of clonazepam plus melatonin treatment, with 3 (23.1%) experiencing clear improvements, 6 (46.1%) partial improvements and 4 (30.8%) reporting no benefits (Table S2). Out of a total 69 RBD patients treated with clonazepam plus add-on therapies other than melatonin, such as carbamazepine, pramipexole, etc., 41 (59.4%) reported clear benefits, 3 (4.4%) partial benefits, while 25 (36.2%) experienced no benefits (Table S3).

Given the strong clinical effectiveness reported for clonazepam there may have been little equipoise to demand comparative studies. Indeed, only a very limited number of prospective comparative studies have been conducted to assess the efficacy of clonazepam to reduce RBD (Table 1). To date, only one RCT (level I-B) tested the efficacy of clonazepam. In this recent study, Shin et al. (2019) compared the clinical effects of 0.5 mg clonazepam treatment to 0.5 mg matched placebo taken before sleep for 4 weeks in a prospectively registered, double-blinded RCT on 20 (active arm) and 20 (placebo arm) PD patients with probable RBD [36]. One patient in the active arm withdrew consent prior to receiving the allocated intervention. The primary outcome was the CGI impression-improvement (CGI-I) score, which is a 7-point ordinal scale, compared between groups at the end of the intervention. Partners, who were instructed to sleep beside the patient for every night of the intervention and record any observed RBD events, were interviewed to assess the CGI-I. Importantly, no differences between groups were found (*p* = 0.253), with subjective RBD severity tending to improve in both groups equally. Also no improvements following clonazepam were noted on any of the secondary subjective sleep severity outcomes as compared to placebo. The combination of a small sample size and a 7-point ordinal outcome measure that is prone to what seems to be powerful placebo effects makes it difficult to confirm the presumed effectiveness of clonazepam for reducing RBD using the outcomes of this single RCT only. The study also lacked PSG recordings to confirm the RBD diagnosis and to objectively assess RWA or DEB severity as a trial outcome [36].

In a recent POS study, Li et al. (2016) prospectively followed 39 iRBD patients taking clonazepam 0.125-3 mg for a mean duration of 28.8 months [37]. The treatment duration was not fixed and only 27 patients remained on the original dosage, while 10 had their prescriptions changed, and 2 were lost to follow-up. There was no control group to compare the treatment effects against and the study was not randomized, nor blinded (Table 1). Interestingly, although a subjective improvement was noted by 26 (66.7%) of the patients, the objective RWA actually worsened over time and no reduction in DEB were noted on PSG [37]. Earlier, Lapierre et al. (1992) prospectively followed 5 iRBD patients, one of whom presented with mild cerebellar signs, taking 0.5-2 mg clonazepam for 2 months [38]. All patients had PSG confirmed RBD and the primary outcomes were phasic and tonic chin EMG activity and DEB during REM sleep recorded with PSG. As a case series it did not employ randomization, blinding, or a control group. The five patients subjectively reported partial improvement of their RBD (Table 1). A reduction in DEB and phasic chin EMG activity during REM sleep was noted, although no reduction in tonic RWA was found [38]. Finally, Iranzo et al. (2005) prospectively followed a group of 39 iRBD, 45 PD, and 26 MSA patients with PSG confirmed RBD who were administered clonazepam, if clinically required, with dosages titrated up until clinical resolution or tolerability [39]. The treatment duration was not fixed with the average follow-up duration being 26.9 months. There was no control group and the study was not randomized, nor blinded. Most patients reported subjective improvements, though no objective outcomes of RBD were compared pre- and post intervention [39].

Two retrospective studies by Ferri et al. (2013a, b) were eligible for inclusion, but as the authors did not report the exact number of clinical responders, this data could not be included in Tables 2 and S1 [11, 12]. Ferri et al. (2013a) first compared the PSGs of 13 iRBD patients before and after they took 0.5–1 mg clonazepam for an average duration of 2.6 ± 1.1 years [11]. The primary outcome was the RBD severity scale (RBDSS), which rates RBD severity based on the DEB recorded by PSG [40], along with the RWA and CGI. No differences were found longitudinally in these patients, indicating that long-term clonazepam administration did not reduce clinical RBD severity. In a second study, Ferri et al. (2013b) retrospectively compared the same outcome measures in a group of 15 iRBD patients assessed before and after taking 0.125–1 mg clonazepam for 2.8 ± 1.6 years. Again, clonazepam was not found to significantly reduce objective or subjective RBD severity [12]. These studies put the presumed magnitude of the clinical effectiveness of clonazepam in doubt. A limitation across these studies is that the data was retrospectively analyzed and that no control intervention was administered to compare the treatment effects against.

### Melatonin

The clinical outcome of melatonin mono-therapy was reported for a total of 137 RBD patients, of whom 45 (32.9%) experienced clear benefits, 37 (27.0%) partial benefits and 55 (40.1%) no benefits (Table S7). This update is based on the clinical outcomes of 3 RCTs [16, 41, 42], 2 POS, 7 RMH, and 10 CRs. Of these, 100 patients were administered immediate release melatonin, with 34% reporting clear benefits, 31% partial benefits and 35% no benefits. A total of 37 patients were administered a prolonged-released formulation of melatonin (Circadin), with 11 (29.7%) reporting clear benefits, 6 (16.2%) partial benefits, and 20 (54.1%) no benefits. Most patients across these studies had secondary diagnoses besides RBD. A combination of melatonin plus an adjunctive therapy other than clonazepam was trialed in just three patients. One patient with iRBD plus palatal tremor with ataxia received melatonin plus ropinirole, which mildly improved RBD [43], and another patient with iRBD received melatonin plus pramipexole, which was unsuccessful until sodium oxybate was added leading to partial resolution of RBD [26] (Table S8). One other iRBD patient received melatonin plus gabapentin, but the effectiveness was not reported [44].

Recently, two double-blinded RCTs (level I-B) with a parallel group design evaluating melatonin were published, one in PD and one in iRBD patients (Table 1). Our trial, Gilat et al. (2019), compared the effects of 4 mg (2 × 2 mg) prolonged-release melatonin (PR-melatonin) to 4 mg (2 × 2 mg) of matched placebo taken 1 h before bedtime for 8 weeks in 30 PD patients (15 per group) with PSG confirmed RBD [42]. The study also had a 4-week extension phase without treatment to test whether melatonin is effective even after you stop taking it as has been previously reported [16]. A patient-centered primary outcome was used, whereby patients and/or bed-partners (if applicable) recorded the frequency and severity of RBD events for each night on a weekly RBD event diary, which they had been trained on for 4 weeks prior to randomization. Importantly, we observed that patients completed their dream enactment diaries on 99% of days. The primary endpoint was the number of documented RBD events per week observed across the last 4 weeks of the treatment period and compared between the groups. Secondary outcomes were the severity of documented RBD events, RWA on PSG, several RBD-related questionnaires including the RBD Questionnaire-Hong Kong (RBDQ-HK), the CGI, as well as 1 week of actigraphy and several other sleep-quality related measures assessed before and during the last 4 weeks of the intervention period. No significant differences were found between the PR-melatonin and placebo groups on any of the RBD-related outcome measures, with both groups improving markedly. Post-hoc analyses revealed that there was no difference in bedtime variability between the groups, suggesting differences in sleep hygiene did not impact on the presumed circadian effectiveness of melatonin. Moreover, sleep onset latencies measured with actigraphy did improve in the melatonin group compared to placebo, in line with the known effects of melatonin [45]. During the 4 week extension phase both the patients originally on melatonin and those on placebo continued to have markedly reduced RBD events compared to baseline and of very similar severity to when they were in the double-blind parts of the study. Limitations of this study were the relatively small groups and that the secondary RWA outcome could only be assessed in a subgroup of the total sample constituting just 14 patients [42].

Around the same time, Jun et al. [41] published their double-blinded RCT (level I-B) using PR-melatonin in adults with PSG confirmed iRBD [41]. They compared three parallel groups, one (*n* = 9) receiving 6 mg (3 × 2 mg) PR-melatonin, one (*n* = 7) receiving 2 mg (1 × 2 mg) PR-melatonin plus 4 mg (2 × 2 mg) matched placebo, and the final arm (n = 9) receiving 6 mg (3 × 2 mg) matched placebo, for 4 weeks of treatment. The primary outcomes were the CGI-I and the Korean version of the RBDQ-HK (RBDQ-KR) compared across groups at the end of treatment. Secondary outcomes included an RBD diary (the outcomes of which were not reported) and subjective sleep quality scales. Again, no significant differences were found between PR-melatonin and placebo groups on subjective RBD or any of the secondary outcomes. There were also no significant improvements observed in any of the groups on the RBDQ-KR or secondary outcomes following the intervention as compared to baseline. Limitations of the study were the small groups and that no objective RBD measures were obtained as an outcome [41]. These two recent RCTs thereby add to the small body of scientific evidence indicating that the presumed clinical effectiveness of first-line RBD treatments may in fact be driven by placebo.

Kunz and Mahlberg [16] conducted a cross-over RCT in eight patients comparing the effects of 4 weeks of 3 mg melatonin to the effects of 4 weeks of 3 mg matched placebo across all subjects, with the order of treatment being randomized [16]. Commonly, RBD patients are instructed to take melatonin 1-h before bedtime, regardless of how variable bedtimes are across nights. A key difference with other trials is that Kunz and Mahlberg [16] instructed their patients to take melatonin at set times between 22.00–23.00 h and to go to bed 30 min after, with the idea that this regime facilitates the chronobiotic effects of melatonin that might lead to reduced RBD [16, 20]. Unfortunately, their trial had to be cut short due to administrative changes in the department and as a result, only eight patients were randomized and completed the study, five of whom had iRBD, one had PD and two had RBD and narcolepsy plus periodic limb movements (PLMS) [16]. The primary outcomes were the number of 3-s mini-epochs of RWA on PSG assessed in a double-blind manner and the CGI compared between treatments at the end of the intervention and for each treatment compared to baseline. Clinically, the authors reported that all, but one patient, reported clear benefits from the melatonin treatment, though the possible benefits following placebo were not reported in a similar vein. When comparing the primary outcomes, the authors noted significant improvements in the number of RWA epochs and the CGI severity score (CGI-S) after melatonin treatment compared to baseline. In addition, the CGI improvement score (CGI-I) was significantly different between groups and judged by the authors to indicate a significant improvement due to melatonin. However, the mean CGI-I after melatonin was 3.3 ± 1.2 and 4.5 ± 0.8 after placebo, whereby a score of 3 on the CGI-I indicates ‘minimal improvement’ and a score of 4 indicates ‘no change’, which might be interpreted as a minor improvement after melatonin compared to placebo. Moreover, when directly comparing the two groups, no significant differences were found for either the number of REM epochs with RWA or CGI-S. Sleep onset latency also significantly improved after both melatonin and placebo [16].

An interesting observation made by the authors was that in the patients receiving the placebo second (*n* = 5), the number of RWA epochs was also significantly lower after placebo as compared to baseline. Based on the idea that the effects of melatonin may outlast the treatment period and the finding that no such improvement was seen in the group receiving placebo first (*n* = 3), the authors interpreted this finding as confirmatory for long-lasting effects of melatonin that carried-over into the second placebo period [16]. However, the comparison done in the group receiving placebo first was severely underpowered (*n* = 3). Furthermore, the 4-week extension period in our own trial [42] indicated that RBD kept improving not only after melatonin, but also after placebo [16]. Future larger RCTs aimed at assessing the efficacy of melatonin for reducing RBD should consider adopting observation periods lasting beyond the intervention period to robustly test this interesting observation.

Two open-label POS studies also assessed the effect of melatonin. Takeuchi et al. [46] classified 13 out of a total of 15 RBD patients receiving 3–9 mg of melatonin as partial responders, though three of them responded remarkably (75% less RBD), while the other 10 indeed responded moderately (50% less RBD) or mildly (25% less RBD) [46]. The treatment duration, and whether the patients had comorbid diagnoses besides their RBD, was not reported. Objectively, melatonin significantly reduced tonic EMG during REM sleep as compared to baseline [46]. During the second PSG on melatonin treatment, blood melatonin concentration levels were sampled every three hours. The authors reported that melatonin concentration was increased in a subset of the patients (exact number not reported) who had low baseline melatonin levels (values not reported) [46]. Kunz and Bes [15] further reported that 3 mg of melatonin for 6 weeks led to substantial clinical improvements in five out of six patients with mixed diagnoses besides their RBD (Table 1) [15]. These clinical effects were considered long-lasting, with clinical responsiveness remaining after treatment cessation, even for as long as 22 months in one patient. Also on PSG, there was a reduction in RWA observed on melatonin as compared to baseline [15]. Given the lack of a control intervention, the outcomes of these open-label studies should be interpreted with caution.

Taken together, to date only three relatively small RCTs and two POS studies have been conducted to test the efficacy of melatonin for reducing RBD. Two of the parallel-group RCTs showed no improvements after melatonin [41, 42] and the third cross-over study, showed partial improvements compared to placebo [16]. These studies thereby highlight the importance of a double-blinded assessment to preclude a seemingly strong placebo effect influencing both the patients and assessors. Importantly, there are much fewer concerns regarding side effects with melatonin compared to clonazepam and for that reason, melatonin is almost certainly a safer first-line treatment option for RBD, especially in the elderly. Based on the current scientific evidence, however, our prior assumption that melatonin has a marked clinical effect should be tempered by the observation of marked placebo and/or regression to the mean effects in placebo-controlled trials. Adequately powered RCTs will provide more precise estimates of the true treatment effect size, if any.

## Alternative treatments for RBD

Eleven other prospective studies were identified that tested the effect of alternative treatments for RBD (see Table 1), including two RCTs on a cholinesterase inhibitor (rivastigmine) [27, 28], one RCT on a glutamatergic antagonist (memantine) [47], two open-label studies on a melatonin-agonist (ramelteon) [48, 49], five open-label studies on dopamine-agonists (pramipexole, ropinirole, and rotigotine) [50–54], and one open-label study on a selective serotonin reuptake inhibitor (paroxetine) [33]. The existing evidence for all other alternative drugs trialed to reduce RBD is based solely on retrospective accounts and case reports (see Table 3). The evidence on the effectiveness of all the alternative treatments for RBD is described in the S*upplementary materials*. Given the lack of robust evidence, to date none of these pharmacological agents can be recommended as first-line treatment options for RBD.

## Publication bias evaluation

A separate search was conducted in the International Clinical Trials Registry Platform of the World Health Organisation to assess for possible publication bias, resulting in 17 eligible trials. Details on each of these trials are tabulated in the Supplementary Materials. The outcomes of three completed RCTs (Registration Identifiers: NCT02836743, NCT02312908 and ACTRN12613000648729), including our own, were published in a peer-reviewed scientific journal and included in the present review [36, 41, 42]. Another trial registration containing limited information (EUCTR-2009-012071-10) is possibly linked to two included publications as they have the same study sponsor and assess the same intervention (4.6 mg rivastigmine patch) [27, 28]. The investigators, however, do not refer to the trial registration in their publications, and some inconsistencies are apparent between the registration and the publications, such as the sample size and primary outcome. Moreover, six recently registered trials are likely still ongoing (i.e., status listed as ‘recruiting’ or ‘not yet recruiting’) and as such could not be assessed for possible publication bias at this time (see Supplementary Table for trial identifiers).

Three listed trials were terminated before the target samples were reached. One RCT on the effects of 8 mg ramelteon compared to placebo was terminated after enrolling only three subjects due to low recruitment rates (NCT00745030), and another open-label trial on the effect of 20–80 mg nelotanserin, a serotonin receptor inverse agonist, was terminated early after changes were made to the overall development program for the study drug (NCT02871427). Our own trial on the effect of 4 mg of PR-melatonin compared to placebo in patients with isolated RBD was also terminated early after enrolling just 6 subjects due to low recruitment rates (ACTRN12613000647730). None of these terminated trials posted any outcome data on the trial registries. Another RCT on the combined effect of clonazepam and melatonin PR with December 2019 as the estimated completion date also has no results listed and has not yet been published (NCT02789592), though the recruitment status of that trial is listed as ‘unknown’, and as such it might still be ongoing. Similarly, a double-blinded trial comparing the effect of melatonin to clonazepam on RBD in PD is listed as ‘completed’, while the results have not been posted nor published (IRCT20170821035819N3). However, that trial was only completed recently in 02/2020, and so the investigators might still be in the process of publishing their findings. Of note is that the registration text, which was posted before the study end date, appears to un-blind the trial investigators.

Importantly, the outcomes of two RCTs that have been completed for over 2 years have also not been published, indicative of possible publication bias. One RCT completed in 2011 tested the effect of 8 mg ramelteon compared to a placebo over 30 nights, but to our knowledge the investigators have not posted nor published the trial results (NCT01401413). Another RCT completed in 2018 on the effect of 40-80 mg nelotanserin compared to placebo over 28 nights in RBD patients with dementia (DLB or PD) has also not been published in a peer-review journal, though the investigators of that trial did disseminate part of the results on the trial registry (NCT02708186). A total of 16 patients (all male) were randomized to receive nelotanserin, and 18 patients (13 males) were randomized to receive matched placebo for 28 days. Two patients in each group dropped-out. The primary outcome was the change in the number RBD events observed on a single night of PSG compared between baseline and post-treatment. Based on an intention-to-treat analysis, the least mean squares (standard error) for the nelotanserin group was − 1.47 (1.006) RBD events and for the Placebo group − 0.26 (1.027). It is not reported whether this finding represents a statistically significant effect. Nelotanserin was also associated with several adverse events. Given the limited amount of evidence, no recommendation can be made for the use of nelotanserin to treat RBD in patients with dementia. Taken together, there is some indication of possible publication bias for pharmacological interventions for RBD.

## Outcomes used

Choosing a primary outcome measure for RBD is challenging [30]. The large majority of studies identified by this literature review relied on subjective recollections from the patient and/or their bed partners to assess the effectiveness of RBD treatments. The 7-point ordinal CGI scale was the most frequently used measure of a clinically evident effect and in some cases a customized scale was devised, such as a three- [33, 53] or four-point [51, 54] ordinal RBD severity rating based on clinical opinion or a VAS scale completed by the bed-partners [48]. However, baseline expectations on the presumed effectiveness of the intervention, as would be the case for first-line treatment options for RBD, create a high risk for bias. Clinical opinions are also at high risk of being influenced by placebo effects, if not controlled for in a double-blinded manner. Furthermore, retrospective recollections of symptom severity can be heavily driven by the occurrence of a single severe event, which may have been an ‘oddball’, rather than an average of all events. Biased recollection may be exacerbated in those RBD patients with memory difficulties, such as those with DLB and PD dementia. People may also struggle to remember whether the RBD events occurred during or outside of the intervention period. Finally, patients with other symptoms besides RBD, for example such as is the case for PD and DLB patients, may report benefits to their clinician after receiving treatment for their RBD, as at that time their desire to resolve RBD may be overshadowed by the desire to resolve some other symptom that may still go untreated. Importantly, all RCTs conducted to date on first-line treatments for RBD included the CGI as either the primary [16, 36, 41] or as a secondary outcome [42]. This ordinal scale makes it difficult to show differences in the small samples that have been studied. Indeed, only one of the RCTs could report a minor improvement on the CGI following the active intervention [16], whereas the three other double-blinded RCTs showed no benefits compared to placebo [36, 41, 42]. While RBD-related questionnaires are useful to screen for the presence of probable RBD, of the questionnaires used by the included RCTs and POS studies only the RBDQ-HK and its Korean version (RBD-KR) were developed to assess RBD severity [55]. As such, the RBDQ-HK or RBDQ-KR was used as either a primary [37, 50] or secondary outcome [42] in three prospective intervention studies. However, RBD-related questionnaires, such as the RBDQ-HK, also rely on subjective retrospective recollections of RBD severity by patients and their bed-partners, questioning their accuracy. Taken together, we would not recommend the exclusive use of subjective retrospective accounts for assessing the efficacy of any RBD intervention in routine clinical practice. The use of CGI should be coupled with sufficient sample sizes to detect meaningful differences in ordinal data and a good randomized double-blinded control (probably placebo at this point).

Two of the recent RCTs on PR-melatonin implemented an RBD event diary as either the primary [42] or as a secondary but unreported outcome [41]. Such patient-centered measures of RBD frequency and severity might be good outcome measures in symptomatic RCTs as long as the subjects are fully blinded to the treatment allocation, and by filling out the diary each morning, there is presumably a reduced risk for recollection error. Diary outcomes measured continuously may thus provide a more sensitive representation of RBD clinical severity (i.e., severe enough and memorable enough to motivate a patient to actually seek clinical help), especially when the entries can be complemented by a bed-partner. We have found the diary seems to have good ‘face’ validity with patients accepting that it looks like it captures patient and bed partners’ complaints [42]. However, the patients themselves are asleep and often the bed-partners are too when RBD occurs, and as such, RBD events may be missed. Moreover, if RBD becomes disruptive of sleep and/or forms a risk for injuries, the bed-partners will often resolve to sleep in a separate room and many RBD patients do not have a bed-partner. Excluding subjects without a bed-partner sleeping in the same room will thus lead to a non-representative sample of the population. Finally, there is no way to control whether the entries provided are accurate. Based on our experience we recommend a training period for patients to learn to adequately complete such an outcome prior to randomization and provide patients with frequent reminders to keep filling out the diary as adequately as possible to prevent missing entries. In our trial such an approach resulted in 99% adherence for completing the primary outcome, ensuring adequate statistical power in the analysis [42].

## Future directions

Our interpretation of the totality of treatment evidence in RBD is that the presumed effectiveness of the two mainstay treatments may be largely or wholly attributable to the non-specific effects of good clinical care, placebo effects, and regression to the mean. As such, it should be a pressing priority in the field today to ascertain how effective the mainstay treatments for symptomatic alleviation truly are. It is time to conduct robustly designed and properly powered and blinded, placebo-controlled parallel group RCTs using outcome measures that are free from interpretation bias.

One of the challenges has been the development of an accurate primary outcome measure of true RBD burden that is specific to RBD and free from subjective interpretation [30]. Actigraphy outcomes have been proposed as an objective outcome for RBD [56, 57], but with actigraphy alone it is impossible to ascertain whether the patient is truly in REM sleep when movements are detected. Similarly, automated 3D video analysis of leg movements during REM sleep, in particular short jerks of 0.1–2.0 s, as captured with a Microsoft Kinect v2 sensor using infrared camera’s was recently shown to be able to accurately (90.4%) distinguish iRBD patients from prodromal RBD and patients with other sleep disorders and leg movements [58]. The number of leg jerks documented with this automated system during REM sleep correlated strongly with RWA and visually scored leg movements. PSG, however, was still required to score REM sleep, in particular as low classification accuracies were reported for non-REM sleep periods [58]. In effect, currently only PSG can objectively detect the presence, frequency and severity of RBD. To date, several RCTs and prospective open-label studies already rated dream enactment behaviors and/or RWA on PSG as an outcome of their intervention [15, 16, 27, 37, 38, 41, 42, 46, 48, 50, 53, 54] with many of these showing no differences (Table 1). However, gold-standard PSG requires an overnight stay in a sleep laboratory, which is costly and involves travel for the patient. A laboratory environment may also be an unfavorable setting for patients to achieve typical sleep, possibly having an impact on the amount of REM sleep. Finally, RBD can be highly variable across nights [40]. As a result, a single night of laboratory PSG may not provide an adequate representation of RBD frequency and severity and this may have precluded past studies from detecting a favorable treatment effect.

Home-based PSG devices (HB-PSG) are now able to collect the same signals as laboratory PSG (i.e., EEG, EOG, nasal flow, thermistor, and importantly EMG), therefore, offering new possibilities for sleep evaluation over multiple nights in the subject’s own homes [59, 60]. Combined with an infrared camera and microphone, such ambulatory PSG devices could, in the near future, offer the same DEB, RBDSS and RWA outcomes as laboratory PSG [59]. They could be conducted without overnight supervision, or trained staff could supervise via remote monitoring [61]. Importantly, the feasibility and validity of HB-PSG has already been demonstrated for the diagnosis and treatment of obstructive sleep apnea (OSA) with surprisingly low failure rates [59, 62]. Specifically, 79% of OSA patients preferred HB-PSG and achieved greater sleep-quality, -efficiency and -duration than during laboratory PSG [60, 62]. The amount of REM sleep was also increased at home compared to laboratory PSG in several studies [60]. Such benefits are of even greater significance in patients with a neurodegenerative disease, such as PD and DLB, who suffer from impaired mobility and heightened sleep sensitivity, especially as multiple testing nights will be required. As such, HB-PSG may serve as a new objective endpoint for future clinical trials for RBD. Thus a next step for the field is to validate HB-PSG by comparing the RBD outcomes to those obtained with laboratory PSG and to determine the natural variability and minimally detectable change of HB-PSG derived RBD outcomes [25].

Of interest is that a home-based screening device was previously evaluated for assessing OSA in PD, showing greater discrepancy in diagnostic accuracy of OSA compared to laboratory PSG [63]. However, PD patients in that study were required to place the sensors on themselves, leading to high failure rates and reduced data quality. In fact, > 15% of subjects declined to participate, because they were not confident about their ability to correctly wear the device [63]. It is, therefore, advised that trained study staff should apply the sensors and conduct system calibration and impedance testing in future HB-PSG studies [59]. Remote monitoring may further help reduce signal loss [61]. Future HB-PSG devices may require fewer sensors and be made easier for patients to apply. Moreover, we recommend incorporating a lead-in period, whereby the HB-PSG is applied during one or preferably several nights to get subjects accustomed to wearing the device and knowledge of being monitored, prior to the randomization, to prevent a regression towards the mean due to a familiarization effect. In addition, care should be taken to prevent weekend-effects, whereby a change in sleep schedule during the weekend may impact on the amount of REM sleep, especially in the working population [64].

High accuracy for detecting the primary outcome measure, such as RWA, short limb jerks, DEB or RBDSS averaged over multiple nights [3, 40, 58] can be ensured through HB-PSG with each RBD event scored on video and confirmed by RWA without OSA-induced arousal. Thereby, HB-PSG systems will provide an objective measure of RBD frequency and severity, which can be obtained in any patient, with- or without- a bed partner, and over multiple nights in the patient’s own homes to maximize the representation of true RBD severity in daily life. Given that complex DEB can be highly variable across nights, perhaps capturing the number of short limb jerks during REM sleep would prove to be a more reliable outcome of overt RBD [3, 58]. Clearly, the costs of such a HB-PSG system and the time needed for trained staff to apply the device and monitor data acquisition represents a potential limitation of this suggested approach.

Another important consideration for future studies is the timing of patient enrollment. Indeed, patients are often enrolled upon first referral to the sleep clinic after they have experienced a period with troublesome RBD symptoms. Given the variability of RBD over time [40], enrollment into a clinical trial during such a period of high RBD severity might result in a regression towards the mean over time, unrelated to the treatment effect. As such, and if clinically ethical to temporarily withhold possibly effective treatment, we recommend future studies to implement an observation period prior to randomization to assess the natural variability in RBD symptom severity, resulting in better statistical power.

A possible limitation of the present review is that we included studies that assessed patients with probable RBD, whose diagnoses were not confirmed by PSG. We also interpreted the clinical effects across all patients with RBD. Future work is needed to determine whether pharmacological effects differ across patient populations, for instance secondary vs. isolated RBD.

## Conclusion

The best current evidence base for pharmacotherapies for RDB could charitably be described as being of an I-b level (*Box 1*). Based on lower levels of evidence, the traditionally claimed effectiveness of the two first-line therapies for RBD (melatonin and clonazepam) may be greatly overestimated. The clinically observed effectiveness of these interventions may have been driven by strong placebo effects, regression towards the mean, and the non-specific but laudable effects of good clinical practice in RBD, such as behavioral advice. Concerns continue to exist about the ability of any outcome measure to accurately and objectively capture RBD severity in an unbiased manner. Thus there is a clear need to conduct more robustly designed and adequately powered double-blind placebo-controlled RCTs using better outcome measures on appropriately selected patient groups. Patient-centered diary outcomes are currently recommended for larger phase 3 trials and following validation, objective RBD as measured by home-based PSG over multiple nights is suggested as the most promising primary endpoint for future earlier phase RCTs on RBD.

## Electronic supplementary material

Below is the link to the electronic supplementary material.

Supplementary file 1 (PDF 89 KB)

Supplementary file 2 (PDF 173 KB)

Supplementary file 3 (PDF 128 KB)
