# Supplementary material for: A critical review of the pharmacological treatment of REM sleep behavior disorder in adults: time for more and larger randomized placebo-controlled trials
Source: J Neurol. 2021 Jan 7;269(1):125–48. doi: 10.1007/s00415-020-10353-0 (PMC8739295; doi:10.1007/s00415-020-10353-0)
Supplement: Supplementary file 1 — (PDF 89 KB) [file 415_2020_10353_MOESM1_ESM.docx]

**Supplementary Materials:**  Brief descriptions of the existing evidence for the alternative treatments trialled to date in older adults to treat RBD

**Title:** An update on the pharmacological treatment of REM sleep behavior disorder in adults: Time for more and larger randomized placebo-controlled trials.

**Authors**: Moran Gilat PhD, Nathaniel Marshall PhD, Dries Testelmans MD PhD, Bertien Buyse MD PhD, Simon JG Lewis MD PhD

**Corresponding author:**

Dr Moran Gilat PhD

Email: [moran.gilat@kuleuven.be](mailto:moran.gilat@kuleuven.be)

**Table of content**

| **Drug Class** | **Drug Type** | **Page** |
| --- | --- | --- |
| Benzodiazepines | Temazepam | 2 |
|  | Zopiclone | 2 |
|  | Other | 2 |
| Melatonin agonist | Ramelteon | 3 |
|  | Agomelatine | 4 |
| Dopamine + agonist | Levodopa | 4 |
|  | Pramipexole | 5 |
|  | Ropinirole | 5 |
|  | Rotigotine | 5 |
| Anticholinergic | Donepezil | 6 |
|  | Rivastigmine | 6 |
| NMDA antagonist | Memantine | 7 |
| Gabapentinoid | Gabapentin | 8 |
|  | Pregabalin | 8 |
| Noradrenergic agonist | Clonidine | 9 |
| Antidepressants (per class) | SSRI | 9 |
|  | Tricyclic | 10 |
|  | Other | 10 |
| Antipsychotics | Mixed types | 11 |
| Anticonvulants | Mixed types | 11 |
| Gamma-hydroxybutyric acid | Sodium Oxybate | 11 |
| Other | Yi-Gan San (Yokukansan) | 12 |
|  | Cannabidiol | 13 |
|  | Cardiac drugs | 13 |
| **References** | | 14 |

1. Benzodiazepines

*1.1 - Temazepam (Table S4)*

Temazepam is an intermediate benzodiazepine often prescribed to aid sleep continuity. It has a shorter 10-hour elimination half-life, compared to 40-hours for clonazepam, though this is still longer than the average sleep duration, especially in the elderly [1]. There are three reports of temazepam use for RBD. One patient with iRBD showed clear improvements [2], while one patient with iRBD and major depression (MD) [3], as well as another RBD patient with a possible condition besides RBD [4], reported no benefits. No comparative studies have been performed for temazepam.

*1.2 - Zopiclone* *(Table S5)*

Zopiclone is a drug of the cyclopyrrolone class, which has similar effects on GABAergic receptors as benzodiazepine drugs. Zopiclone has a much shorter 5-hour elimination half-life, and has therefore been proposed for RBD in order to limit hangover effects the next morning [4]. The clinical effects of zopiclone were reported for a total of 12 patients, two with iRBD [2, 5], one with iRBD plus MD [3] and 9 with mixed conditions [4]. Of these, 7 (58.3%) reported clear benefits, while 5 (41.7%) reported no benefits (*Table S12*). No comparative studies have been performed for zopiclone.

*1.3 - Other benzodiazepines (Table S6)*

Anderson et al. (2009) achieved symptom control in one RBD patient who was refractory to different kinds of monotherapy by administering a combination of temazepam plus zopiclone [4]. However, the dosage and duration of the treatment were not reported nor was it reported whether this patient had any other condition besides RBD. Triazolam treatment was trialed by Olson et al. (2000) in two RBD patients refractory to clonazepam and with unknown conditions besides RBD [6]. One of them reported clear benefits whereas the results in the other were uncertain. Fernandez-Acros et al. (2016) reported that 14 patients with mixed conditions besides RBD had no benefit from benzodiazepines other than clonazepam, without reporting which drugs were trialled or for how long [7]. Shinno et al. (2008) reported no benefits in 1 iRBD patient with either 5mg of nitrazepam or bromazepam treatment [8]. Schenck et al. (1986) reported that two patients with iRBD did not benefit from alprazolam 0.5mg [9]. Finally, Escriba et al. (2016) reported to have effectively treated RBD using benzodiazepines 0.5-2mg in one RBD patient with an unknown condition, though they did not report which drugs were administered and for how long [10]. Out of a total of 22 patients, 86.4% reported no benefits. No comparative studies have been performed.

2. Melatonin agonists

*2.1 - Ramelteon (Table S9)*

Ramelteon is a melatonin MT_1_ and MT_2_ receptor agonist with selectivity for MT_3_ receptors as well. It gained particular interest in Japan where melatonin is not a formally approved drug, whereas ramelteon got approved for treatment of insomnia [11]. Kashihara et al. (2016) noted a significant reduction on the Japanese version of the RBD screening questionnaire (RBDQ-JP) across 24 PD patients with probable RBD who took 8mg of ramelteon for 12 weeks as compared to baseline, though surprisingly a reduction on the RBDQ-JP was also seen in another 11 PD patients without probable RBD [12]. The exact number of responders was not reported and the study was open-label. Another prospective open-label study further showed that 10 out of 12 patients with iRBD reported no benefits from 8mg ramelteon and also no clear improvements on RSWA or RBSS on PSG were found [11]. The treatment duration was not reported. Two earlier case-reports (*Table S9*) noted beneficial effects from 8mg of ramelteon to reduce RBD in 2 DLB patients [13], one MSA patient, and one PD patient [14]. Across the 16 patients with RBD for whom clinical responsiveness was reported, 5 (31.25%) indicated clear benefits, 1 partial benefits (6.25%), and 10 no benefit (62.5%) (*Table S9*). Only mild adverse effects were noted in 8 out of the 51 patients taking ramelteon (including patients without RBD), rendering it a relatively safe treatment option to trial in patients for whom melatonin is not available, though the effectiveness appears limited and a placebo-controlled RCT is lacking as of yet.

*2.2 - Agomelatine (Table S10)*

Agomelatine is an atypical antidepressant, which act as a melatonin (MT_1_ and MT_2_) agonist and a serotonin receptor antagonist. Bonakis et al. (2012) describe three drug-naïve patients with PSG confirmed iRBD who reported to benefit from agomelatine (25-50mg) treatment for up to 6 months of follow-up [15]. Dream content was also reported to have become more pleasant. A repeated PSG after 6 months of treatment revealed a reduction in tonic RSWA in 2 out of the 3 patients, though this could not be statistically compared and there was no control condition [15]. No comparative studies have been performed.

3. Dopamine + agonist

*3.1 - Levodopa (Table S11)*

Levodopa is the mainstay dopamine replacement treatment for people with PD and highly effective to reduce cardinal motor symptom severity. Evening dose or controlled-release levodopa administration overnight may also reduce nocturnal akinesia, which could indirectly lead to improved sleep quality [16]. It follows that the effect of levodopa on RBD has been mainly tested in PD patients, but so far only in three retrospective studies and 1 case report. Fernandez-Acros et al. (2016) noted that 1 patient with RBD did not benefit from levodopa-carbidopa [7]. The diagnosis of the patient and treatment dosage used were not reported. Bonakis et al. (2009A) reported that 3 patients with PD and 3 patients with iRBD benefited from levodopa treatment, though the dose or duration was not reported [2]. Tan et al. (1996) similarly reported a subjective benefit in 3 PD patients with probable RBD after levodopa, though again the dose and duration were not reported [17]. The largest sample was reported by Özekmeçi et al. (2005), who in contrast to the other reports, noted that levodopa 460±250mg did not prevent the occurrence of RBD in 35 PD patients with probable RBD, 12 of whom were taking levodopa monotherapy and 25 levodopa plus a dopamine-agonist (pergolide, lisuride or bromocriptine) [18]. No objective RBD outcomes were obtained and there was no control condition in any of the studies. Given that the large majority of PD patients are taking levodopa treatment while RBD is still frequently troublesome, makes it unlikely that levodopa mono-therapy is sufficient to treat RBD in these patients. No comparative studies have been performed.

*3.2 - Dopamine agonist (Tables S12-S14)*

Pramipexole is a dopamine D_2_, D_3_, and D_4_ receptor agonist that is frequently used to treat PD motor symptoms, as well as restless legs syndrome (RLS) and periodic limb movements (PLMS). Sasai et al. (2012) noted a subjective improvement in RBD in 12 out of 15 iRBD patients with PLMS of whom 10 also reported a reduction in disturbed dreaming [19]. No change in RSWA was noted on pramipexole as compared to baseline. The treatment dosage and duration were not fixed and the study was rated to be of poor quality (*Table 1*). Similarly, Fantini et al. (2003) reported that 5 out of 8 iRBD patients benefitted from pramipexole as based on subjective rating [20]. They also noted reduced DEB on PSG, though surprisingly the RSWA was increased on pramipexole treatment compared to baseline. The treatment duration was also not fixed [20]. Fernandez-Acros et al (2016) reported pramipexole (dosage unknown) did not reduce RBD in 1 patient with a possible secondary diagnosis (not reported) [7]. Kumru et al. (2008) further showed that the addition of pramipexole 0.54mg per night for 3 months did not resolve subjective or objective RBD in 11 PD patients with RBD on otherwise levodopa monotherapy [21]. The large majority of patients (n=81) reported to date, however, were iRBD cases who’s medical histories were retrospectively studied by Sasai et al. (2013) [22], while one other case study reported the clinical effectiveness of pramipexole of another 10 patients with mixed conditions besides their RBD [23]. Taken together, the clinical effect of pramipexole was reported for a total of 126 patients, with 56.3% reporting clear benefits, 3.2% partial benefits and 40.5% no benefits (*Table S12*).

Ropinirole is another dopamine D_2_, D_3_, D_4_ receptor agonist used to treat PD and RLS. It has been trialed for RBD in just three studies, namely one prospective open-label study whereby 5 PD patients reported no benefit from a prolonged-release formulation [24], one retrospective study reporting that one RBD patient did not benefit (dosage and formulation not reported) [7], and one case report on an iRBD patient plus palatal tremor with ataxia [25] reporting partial benefit from an immediate release formulation (*Table S13*).

A rotigotine patch (12.4±4.3mg) was reported to be clinically effective in 7 out of 11 PD patients in a prospective open-label study, though no differences in DEB or RSWA were noted on PSG [26] (*Table S14*). This open-label level II-B study was also rated to be of poor quality (*Table 1*). Taken together, dopamine agonists have offered mixed results for reducing RBD. No RCT has been conducted for dopamine agonists.

4. Anticholinergic

*4.1 - Donepezil (Table S15)*

Donepezil is an acetylcholinesterase inhibitor commonly prescribed to support mental functions in people with dementia, such as Alzheimer’s disease (AD) or DLB. It gained interest after Ringman et al. (2000) reported three patients, one young adult, one DLB patient and one patient with probable AD, experienced partial improvement of their RBD after taking donepezil 10-15mg [27]. After this, donepezil was trialed in two other case reports on 4 DLB patients of whom 1 reported clear benefit [28], 1 partial benefit [29], and 2 no benefit [28]. Finally, Boeve et al. (2003) provided an anecdotal account that in their clinical experience with over 50 DLB patients, none reported an improvement of RBD after donepezil treatment [30]. As such, only 1.8% of cases published to date reported a clear benefit, 5.4% a partial benefit and 92.8% no benefit from donepezil for reducing RBD. No comparative studies have been performed.

*4.2 - Rivastigmine (Table S16)*

Rivastigmine is another acetylcholinesterase inhibitor prescribed to support mental functions in people with dementia. Two RCT’s and one case report have trialed rivastigmine to treat RBD. Brunetti et al. (2014) conducted a single (patient)-blinded RCT with a cross-over design to assess whether 30 days of 4.6mg rivastigmine per 24hours (patch) would reduce RBD, as compared to a similar period of matched placebo, in 25 patients with mild cognitive impairment and PSG confirmed RBD who were considered refractory to first-line clonazepam and melatonin treatments [31]. No objective outcomes were obtained, though the primary outcome was RBD frequency as recorded on an event diary by the bed-partners. Rivastigmine significantly reduced RBD frequency as compared to placebo, with 18 patients being considered as responders (≤50% reduction in RBD frequency) [31]. Di Giacopo et al. (2012) conducted a similar, but double-blinded, crossover RCT to assess the effect of 4.6mg rivastigmine per 24 hours (patch) for 3 weeks over a matched placebo for 3 weeks in 12 PD patients with PSG confirmed RBD who were also refractory to first-line treatment options [32]. Two patients dropped-out, so treatment outcomes were reported for a total of 10 patients. The primary outcome was RBD frequency as noted on a dairy by the bed-partners, while objective RSWA on PSG was obtained pre- and post the intervention periods for a subset of 4 patients. A significant reduction in RBD frequency was found on rivastigmine compared to placebo, and the reduction was more consistent in patients with greater RBD frequencies at baseline. No change in RSWA was noted in the subset of 4 patients with repeated PSG’s. Seven patients could be considered as responders (>50% reduction), 1 partial responder and 2 non-responders [32]. Importantly, however, a case study by Yeh et al. (2010) reported that rivastigmine might have induced RBD in a patient with AD [33]. Taken together, 69.4% of RBD patients reported benefits, 2.8% partial benefits, and 27.8% no benefit from rivastigmine. However, as there is at least one other report of rivastigmine possibly inducing RBD in a patient with probable AD [34], cholinesterase inhibitors should only be trailed in patients who are refractory to first-line treatments and great care should be taken not to induce or worsen RBD in patients with dementia.

5 - NMDA antagonist *(Memantine)*

Larsson et al. (2010) reported a secondary outcome related to RBD, which was obtained from a previously published double-blinded RCT on the effectiveness of 24 weeks of 5-20mg memantine, a glutamatergic *N*-methyl-D-aspartate receptor antagonist, for treating dementia in people with PD or DLB, as compared to matched placebo [35]. One of the outcomes of that trial was the Stavanger Sleep Questionnaire, which contains a single question addressing probable RBD, namely “*Is the patient physically active during sleep?*” with possible answers being either no, mild, moderate or severe [35]. A total of 27 patients were randomized to memantine, and 30 to placebo. Ten patients dropped-out, leaving 25 in the memantine group and 22 in the placebo at the end of the study. At baseline, the overall frequency of probable RBD was 54%. The exact number of responders was not reported, though the authors noted that the number of patients reporting no or only mild probable RBD increased over time, while the number of patients reporting moderate probable RBD decreased over time in the memantine group, but not in the placebo group. There was also a significant between-group difference at the end of the intervention, indicating a favorable effect for memantine over placebo [35]. However, it is important to note that this study is based solely on secondary outcomes from a previously published RCT, with the RBD-related outcome being a single questionnaire item on physical activity during sleep, which may, or may not have been specific to RBD [35]. Therefore, no recommendation for memantine can be made based on the outcomes of this study alone.

6. Gabapentinoid *(Tables S17-S18)*

Gabapentin is an anti-epileptic drug used to prevent seizures or treat nerve pain. A case study on a single iRBD patient with MD reported no benefits from gabapentin on RBD [3]. Another patient with a possible condition besides RBD (unreported), and who may have been <50 years of age (unreported), also did not benefit from gabapentin in a retrospective observational study [4]. Neither of these studies reported the dosage used. A retrospective study by Escriba et al. (2016) reported benefits of 300-800mg gabapentin in 12 out of 14 patients with mixed conditions besides RBD, though the other 2 patients reported no benefits [10]. The same authors also reported that 2 out of 3 of their patients with mixed conditions besides RBD benefited from 75-150mg pregabalin, which is another drug of the gabapentinoid class, while the third patient did not benefit [10]. Gabapentinoids have several possibly serious side effects associated to them and their use should therefore be monitored with great care. Taken together, there is insufficient evidence to recommend these drugs for treating RBD, and if trialled, the patient should be monitored carefully.

7. Noradrenergic agonist *(Table S19)*

Clonidine is a selective partial receptor agonist for central and peripheral noradrenaline-releasing neurons, by which clonidine stimulation inhibits noradrenaline release. It was shown to reduce REM sleep time and phasic EMG activity during REM sleep in healthy adults [5, 36]. It was thus hypothesized that clonidine would directly influence the maintenance of REM sleep muscle atonia via noradrenergic transmission in patients with RBD [5]. Only two cases of clonidine use are reported. Nash et al. (2003) reported one iRBD patient who benefited from a treatment plan of one week with clonidine (100-200μg) interspersed by one week without treatment [5], while Shneerson et al. (2009) noted no benefit from clonidine in a single iRBD patient with MD (dose and duration not reported) [3]. No objective RBD outcomes were assessed and there was no control condition. Importantly, there is a report of mirtazapine, which is an presynaptic receptor antagonist leading to increased noradrenergic neurotransmission, inducing RBD in 4 patients with PD [37], which together with the clinical response of a noradrenergic agonist in at least one iRBD patient may suggest that the noradrenergic circuit is implicated in the pathophysiology of RBD [5]. However, the current body of evidence is too limited to make a risk-benefit assessment for the use of noradrenergic agonists for treating RBD.

8. Antidepressants (per class)

There have been several case reports [38] and retrospective studies [39] indicating that antidepressant drugs are associated with an increased risk-ratio for inducing or aggravating RBD symptoms. The clinical evidence shown below from studies attempting to treat RBD with such drugs should therefore be interpreted with caution.

*8.1 - SSRI (Table S20)*

The only prospective study conducted to date on any antidepressant or antipsychotic drug tested the effect of paroxetine (10-40mg), a selective serotonin-reuptake inhibitor (SSRI), for reducing RBD in 19 patients with PSG confirmed iRBD presumably by reducing the amount of REM sleep

[40]. The study was open-label and there was no control condition (*Table 1*). Sixteen of the iRBD patients reported partial improvements, whereas severe RBD persisted in three patients. Side effects were noted, including nausea, dizziness, and diarrhea, which led to treatment cessation in two patients

[40]. Two other case studies also reported mixed results for paroxetine, with one iRBD patient reporting partial benefits with 10mg [41], whilst another reported no benefits with 20mg [8]. Two case studies further reported that fluvoxamine (50mg) [41] and trazodone (dosage not reported) [5], both SSRI’s, did not improve RBD in single iRBD patients. A final case study reported no benefits following sertraline (100-150mg) treatment in an RBD patient with OSA, major depression and mild cognitive impairment [42]. Taken together, across a total of 24 patients with RBD, SSRI’s led to partial benefits in 70.8% and no benefits in 29.2%. No comparative studies have been conducted for SSRI’s, whilst there are reports of SSRI’s aggravating RBD [38], indicating these drugs are not favourable for treating RBD.

*8.2 - Tricyclic antidepressants (Table S21)*

One case study reported beneficial effects following carbamazepine 100mg reduced RBD in a single iRBD case [43], while a restrospective account on another patient reported no benefit from carbamazepine (dosage not reported) [7]. Fernandez-Acros et al. (2016) further reported that another patient did not benefit from imipramine (dosage not reported) [7]. Yet another case study reported that desipramine (50-250mg) was also not effective in two iRBD patients [9]. No benefits were also reported following amitriptyline in two iRBD patients reported in two case studies, one administering 50 mg [5], and one not reporting the dosage used [9]. Similarly, clomipramine (100mg) was not effective in a single iRBD patient [44] and dothiepin (150mg) did not reduce RBD in a single patient with RBD, MD and MCI [42]. Taken together, 8 out of 9 patients reported no benefits from tricyclic antidepressants. No comparative studies have been conducted.

*8.3 - Other antidepressants* *(Table S22)*

One case study reported that nefazodone (dosage not reported), an atypical serotonin antagonist and reuptake inhibitor, was not effective in a single iRBD patient [5]. Similarly, mianserin (10mg) [41], a tetracyclic antidepressant, as well as venlafaxine (dosage not reported) [5], a serotonin-norepinephrine-dopamine reuptake inhibitor, were reported not to be effective in two single iRBD patients. Critically, tandospirone, an antidepressant drug of the azapirone class, was shown to aggravate RBD symptoms in a single iRBD patient [41], and mirtazapine, a noradrenergic and specific serotonin antagonist (NaSSA) was shown to induce RBD in four PD patients with RBD [37]. Taken together, none of the 8 patients in total reported benefits, while antidepressants can aggravate or induce RBD in some patients. No comparative studies have been conducted.

9. Antipsychotics (*Table S23*)

Clozapine, an atypical antagonist that binds to serotonin and dopamine receptors and may interact with GABA receptors, was reported to be beneficial for RBD in two patients with dementia and partially effective in another patient with RBD and dementia [6, 45]. The dosages and durations of the intervention were not reported. Quetiapine 25mg, another atypical antagonist of serotonin, dopamine and norepinephrine receptors, was reported to be beneficial in one patient with a possible condition besides RBD (not reported) [46], while the same dosage was not effective in two cancer patients with RBD [47]. Haloperidol, a butyrophenone type antipsychotic, was reported not to be effective in three patients, one with RBD and dementia [48], one with RBD and a possible secondary condition (not reported) [7], and one with RBD and cancer [47]. No comparative studies have been conducted for any of the antipsychotics.

10. Anticonvulants (*Table S24*)

A retrospective account by Fernandez-Acros et al. (2016) indicates that 3 patients with RBD did not benefit from anticonvulants, namely phenobarbital, lamotrigine, or oxcarbazepine [7]. The diagnosis of the patients, dosages used, and treatment durations were not reported. No comparative studies exist.

11. Sodium Oxybate *(Table S25))*

Sodium oxybate (SO) influences gamma-aminobutyric acid-B receptors and is used to form gamma-hydroxybutyric acid (GHB). Despite the risk for illicit misuse, SO is a registered drug for treating cataplexy and excessive somnolence in narcolepsy patients [49], possibly by enhancing slow-wave EEG during sleep, though it also leads to hypothermia and hypolocomotion [50] and may alter dream mentation [49]. In animal models, low dosage of SO inhibits dopamine signalling, while this is increased at higher dosages [50, 51]. To date, five clinical cases have been published whereby SO was administered to treat five patients with mixed conditions besides RBD. One study did not report the dosage used, whilst the dosage in the other 4 patients ranged from 4.5-6mg. SO was considered highly effective in all patients. Mogdaham et al. (2017) treated two iRBD patients, one of whom received melatonin 5mg plus pramipexole 0.45mg plus 4.5mg SO [49]. No worsening in symptom severity occurred after removal of melatonin, and hence the final treatment consisted of pramipexole plus SO. The other patient first received clonazepam 2mg plus melatonin 5mg, and next clonazepam 2mg plus pramipexole 0.36mg, both resulting only in temporal improvement. The patient was thus considered refractory to melatonin and pramipexole, and SO was later added to the clonazepam resulting in a sustained reduction of RBD [49]. Overall, the mechanism of action for SO remains elusive and no comparative studies have been conducted to assess the true efficacy of SO for reducing RBD. Particular care should be taken when attempting to treat PD patients with SO, given the possible influence on dopamine signalling.

11. Other drugs

*11.1 - Yi-Gan San /Yokukansan (Table S26)*

Yi-Gan San, otherwise known as Yokukansan, is an herbal medicine containing a mixture of herbal ingredients [8]. In Japan, Yi-Gan San is registered for treating insomnia, though its mechanisms of action for modulating sleep remain elusive [8, 52]. Shinno et al. (2008) first reported beneficial effects of 2.5mg Yi-Gan San for treating RBD in a patient with PSG confirmed iRBD [8]. Matsui et al. (2019) then retrospectively analysed the outcomes of Yi-Gan San in 36 iRBD patients, 17 of whom received monotherapy and 19 received add-on clonazepam and/or pramipexole (exact number of patients, treatment schemes and dosages not reported) besides Yi-Gan San [52]. Treatment response was assessed with the CGI scale. The outcomes of their study indicated that 12 out of 17 patients on monotherapy and 4 out of 19 receiving add-on therapy reported clear benefits [52]. Taken together, 45.9% of iRBD patients reported benefits from Yi-Gan San treatment, though no comparative studies exist to date.

*11.2 - Cannabidiol (Table S27)*

Chagas et al. (2014) published the only case series to date on the use of cannabidiol for treating RBD [53]. They presented the secondary outcomes from an RCT aimed at assessing the effect of cannabidiol to reduce psychosis in PD. After breaking the blind, the authors found that four of the PD patients enrolled in the cannabidiol group had probable RBD, which was confirmed with PSG in two. Three of them received 75mg of cannabidiol and one received 300mg for 6 weeks. RBD severity was clinically assessed by a neurologist specialized in sleep disorders. All four patients reported prompt and substantial improvements in their RBD after cannabidiol treatment [53]. No other comparative study exists and further research into the effectiveness and underlying mechanisms of this seemingly safe treatment option is needed.

*11.3 - Cardiac drugs (Table S28)*

Schenck et al. (1987) reported that a physician, other than the authors themselves, unsuccessfully attempted to treat RBD in an iRBD patient with metropolol and/or aspirin (dosages not reported) before referring the patient to the sleep clinic [48]. Currently, no indication exists that these drugs would reduce RBD.

**References**

1. Moraes W, Piovezan R, Poyares D, et al (2014) Effects of aging on sleep structure throughout adulthood: a population-based study. Sleep Med 15:401–409. doi: 10.1016/j.sleep.2013.11.791

2. Bonakis A, Howard RS, Ebrahim IO, et al (2009) REM sleep behaviour disorder (RBD) and its associations in young patients. Sleep Med 10:641–645. doi: 10.1016/j.sleep.2008.07.008

3. Shneerson JM (2009) Successful treatment of REM sleep behavior disorder with sodium oxybate. Clin Neuropharmacol 32:158–159. doi: 10.1097/WNF.0b013e318193e394

4. Anderson KN, Shneerson JM (2009) Drug treatment of REM sleep behavior disorder: the use of drug therapies other than clonazepam. J Clin Sleep Med 5:235–239.

5. Nash JR, Wilson SJ, Potokar JP, Nutt DJ (2003) Mirtazapine induces REM sleep behavior disorder (RBD) in parkinsonism. Neurology 61:1161–author reply 1161. doi: 10.1212/wnl.61.8.1161

6. Olson EJ, Boeve BF, Silber MH (2000) Rapid eye movement sleep behaviour disorder: demographic, clinical and laboratory findings in 93 cases. Brain 123 ( Pt 2):331–339. doi: 10.1093/brain/123.2.331

7. Fernández-Arcos A, Iranzo A, Serradell M, et al (2016) The Clinical Phenotype of Idiopathic Rapid Eye Movement Sleep Behavior Disorder at Presentation: A Study in 203 Consecutive Patients. Sleep 39:121–132. doi: 10.5665/sleep.5332

8. Shinno H, Kamei M, Nakamura Y, et al (2008) Successful treatment with Yi-Gan San for rapid eye movement sleep behavior disorder. Prog Neuropsychopharmacol Biol Psychiatry 32:1749–1751. doi: 10.1016/j.pnpbp.2008.06.015

9. Schenck CH, Bundlie SR, Ettinger MG, Mahowald MW (1986) Chronic behavioral disorders of human REM sleep: a new category of parasomnia. Sleep 9:293–308. doi: 10.1093/sleep/9.2.293

10. Escribá J, Hoyo B (2016) Alternatives to Clonazepam in REM Behavior Disorder Treatment. J Clin Sleep Med 12:1193–1193. doi: 10.5664/jcsm.6068

11. Esaki Y, Kitajima T, Koike S, et al (2016) An Open-Labeled Trial of Ramelteon in Idiopathic Rapid Eye Movement Sleep Behavior Disorder. J Clin Sleep Med 12:689–693. doi: 10.5664/jcsm.5796

12. Kashihara K, Nomura T, Maeda T, et al (2016) Beneficial Effects of Ramelteon on Rapid Eye Movement Sleep Behavior Disorder Associated with Parkinson's Disease - Results of a Multicenter Open Trial. Intern Med 55:231–236. doi: 10.2169/internalmedicine.55.5464

13. Kasanuki K, Iseki E, Nishida Y, et al (2013) Effectiveness of ramelteon for treatment of visual hallucinations in dementia with Lewy bodies: a report of 4 cases. J Clin Psychopharmacol 33:581–583. doi: 10.1097/JCP.0b013e318295fdf4

14. Nomura T, Kawase S, Watanabe Y, Nakashima K (2013) Use of ramelteon for the treatment of secondary REM sleep behavior disorder. Intern Med 52:2123–2126. doi: 10.2169/internalmedicine.52.9179

15. Bonakis A, Economou N-T, Papageorgiou SG, et al (2012) Agomelatine may improve REM sleep behavior disorder symptoms. J Clin Psychopharmacol 32:732–734. doi: 10.1097/JCP.0b013e31826866f8

16. Loddo G, Calandra-Buonaura G, Sambati L, et al (2017) The Treatment of Sleep Disorders in Parkinson's Disease: From Research to Clinical Practice. Front Neurol 8:42. doi: 10.3389/fneur.2017.00042

17. Tan A, Salgado M, Fahn S (1996) Rapid eye movement sleep behavior disorder preceding Parkinson's disease with therapeutic response to levodopa. Mov Disord 11:214–216. doi: 10.1002/mds.870110216

18. Ozekmekçi S, Apaydin H, Kiliç E (2005) Clinical features of 35 patients with Parkinson's disease displaying REM behavior disorder. Clin Neurol Neurosurg 107:306–309. doi: 10.1016/j.clineuro.2004.09.021

19. Sasai T, Inoue Y, Matsuura M (2012) Effectiveness of pramipexole, a dopamine agonist, on rapid eye movement sleep behavior disorder. Tohoku J Exp Med 226:177–181.

20. Fantini ML, Gagnon J-F, Filipini D, Montplaisir J (2003) The effects of pramipexole in REM sleep behavior disorder. Neurology 61:1418–1420. doi: 10.1212/wnl.61.10.1418

21. Kumru H, Iranzo A, Carrasco E, et al (2008) Lack of effects of pramipexole on REM sleep behavior disorder in Parkinson disease. Sleep 31:1418–1421.

22. Sasai T, Matsuura M, Inoue Y (2013) Factors associated with the effect of pramipexole on symptoms of idiopathic REM sleep behavior disorder. Parkinsonism and Related Disorders 19:153–157. doi: 10.1016/j.parkreldis.2012.08.010

23. Schmidt MH, Koshal VB, Schmidt HS (2006) Use of pramipexole in REM sleep behavior disorder: results from a case series. Sleep Med 7:418–423. doi: 10.1016/j.sleep.2006.03.018

24. Dusek P, Bušková J, Růžička E, et al (2010) Effects of ropinirole prolonged-release on sleep disturbances and daytime sleepiness in Parkinson disease. Clin Neuropharmacol 33:186–190. doi: 10.1097/WNF.0b013e3181e71166

25. Bonakis A, Papageorgiou SG, Merritt S, Williams AJ (2009) REM behaviour disorder preceding palatal tremor. Sleep Med 10:1161–1163. doi: 10.1016/j.sleep.2009.04.007

26. Wang Y, Yang Y, Wu H, et al (2016) Effects of Rotigotine on REM Sleep Behavior Disorder in Parkinson Disease. J Clin Sleep Med 12:1403–1409. doi: 10.5664/jcsm.6200

27. Ringman JM, Simmons JH (2000) Treatment of REM sleep behavior disorder with donepezil: a report of three cases. Neurology 55:870–871. doi: 10.1212/wnl.55.6.870

28. Massironi G, Galluzzi S, Frisoni GB (2003) Drug treatment of REM sleep behavior disorders in dementia with Lewy bodies. Int Psychogeriatr 15:377–383. doi: 10.1017/s1041610203009621

29. Ozaki A, Nishida M, Koyama K, et al (2012) Donepezil-induced sleep spindle in a patient with dementia with Lewy bodies: a case report. Psychogeriatrics 12:255–258. doi: 10.1111/j.1479-8301.2012.00411.x

30. Boeve BF, Silber MH, Ferman TJ (2003) Melatonin for treatment of REM sleep behavior disorder in neurologic disorders: results in 14 patients. Sleep Med 4:281–284.

31. Brunetti V, Losurdo A, Testani E, et al (2014) Rivastigmine for refractory REM behavior disorder in mild cognitive impairment. Curr Alzheimer Res 11:267–273.

32. Di Giacopo R, Fasano A, Quaranta D, et al (2012) Rivastigmine as alternative treatment for refractory REM behavior disorder in Parkinson's disease. Mov Disord 27:559–561. doi: 10.1002/mds.24909

33. Yeh S-B, Yeh P-Y, Schenck CH (2010) Rivastigmine-induced REM sleep behavior disorder (RBD) in a 88-year-old man with Alzheimer's disease. J Clin Sleep Med 6:192–195.

34. Carlander B, Touchon J, Ondze B, Billiard M (1996) REM sleep behavior disorder induced by cholinergic treatment in Alzheimer's disease. J Sleep Res 5:28.

35. Larsson V, Aarsland D, Ballard C, et al (2010) The effect of memantine on sleep behaviour in dementia with Lewy bodies and Parkinson's disease dementia. Int J Geriatr Psychiatry 25:1030–1038. doi: 10.1002/gps.2506

36. Gentili A, Godschalk MF, Gheorghiu D, et al (1996) Effect of clonidine and yohimbine on sleep in healthy men: a double-blind, randomized, controlled trial. Eur J Clin Pharmacol 50:463–465. doi: 10.1007/s002280050141

37. Onofrj M, Luciano AL, Thomas A, et al (2003) Mirtazapine induces REM sleep behavior disorder (RBD) in parkinsonism. Neurology 60:113–115. doi: 10.1212/01.wnl.0000042084.03066.c0

38. Schenck CH, Mahowald MW, Kim SW, et al (1992) Prominent eye movements during NREM sleep and REM sleep behavior disorder associated with fluoxetine treatment of depression and obsessive-compulsive disorder. Sleep 15:226–235. doi: 10.1093/sleep/15.3.226

39. Teman PT, Tippmann-Peikert M, Silber MH, et al (2009) Idiopathic rapid-eye-movement sleep disorder: associations with antidepressants, psychiatric diagnoses, and other factors, in relation to age of onset. Sleep Med 10:60–65. doi: 10.1016/j.sleep.2007.11.019

40. Yamamoto K, Uchimura N, Biological MHSA, 2006 (2006) Evaluation of the effects of paroxetine in the treatment of REM sleep behavior disorder. Wiley Online Library 4:190–192. doi: 10.1111/j.1479-8425.2006.00212.x

41. Takahashi T, Mitsuya H, Murata T, et al (2008) Opposite effects of SSRIs and tandospirone in the treatment of REM sleep behavior disorder. Sleep Med 9:317–319. doi: 10.1016/j.sleep.2007.05.003

42. Clarke NA, Williams AJ, Kopelman MD (2000) Rapid eye movement sleep behaviour disorder, depression and cognitive impairment. Case study. Br J Psychiatry 176:189–192. doi: 10.1192/bjp.176.2.189

43. Bamford CR (1993) Carbamazepine in REM sleep behavior disorder. Sleep 16:33–34.

44. Chung KF, Wong MT (1994) Rapid eye movement sleep behaviour disorder in a Chinese male. Aust N Z J Psychiatry 28:144–146. doi: 10.3109/00048679409075857

45. Boeve BF, Silber MH, Ferman TJ, et al (1998) REM sleep behavior disorder and degenerative dementia: an association likely reflecting Lewy body disease. Neurology 51:363–370. doi: 10.1212/wnl.51.2.363

46. Lin F-C, Lai C-L, Huang P, et al (2009) The rapid-eye-movement sleep behavior disorder in Chinese-Taiwanese patients. Psychiatry Clin Neurosci 63:557–562. doi: 10.1111/j.1440-1819.2009.01998.x

47. Shinno H, Kamei M, Maegawa T, et al (2010) Three patients with cancer who developed rapid-eye-movement sleep behavior disorder. J Pain Symptom Manage 40:449–452. doi: 10.1016/j.jpainsymman.2010.01.016

48. Schenck CH, Bundlie SR, Patterson AL, Mahowald MW (1987) Rapid eye movement sleep behavior disorder. A treatable parasomnia affecting older adults. JAMA 257:1786–1789.

49. Moghadam KK, Pizza F, Primavera A, et al (2017) Sodium oxybate for idiopathic REM sleep behavior disorder: a report on two patients. Sleep Med 32:16–21. doi: 10.1016/j.sleep.2016.04.014

50. Mayer G (2016) Efficacy of sodium oxybate on REM sleep behavior disorder in a patient with narcolepsy type 1. Neurology 87:2594–2595. doi: 10.1212/WNL.0000000000003389

51. Bustos G, Kuhar MJ, Roth RH (1972) Effect of gamma-hydroxybutyrate and gamma-butyrolactone on dopamine synthesis and uptake by rat striatum. Biochem Pharmacol 21:2649–2652. doi: 10.1016/0006-2952(72)90233-x

52. Matsui K, Sasai-Sakuma T, Ishigooka J, et al (2019) Effect of Yokukansan for the Treatment of Idiopathic Rapid Eye Movement Sleep Behavior Disorder: A Retrospective Analysis of Consecutive Patients. J Clin Sleep Med 15:1173–1178. doi: 10.5664/jcsm.7816

53. Chagas MHN, Eckeli AL, Zuardi AW, et al (2014) Cannabidiol can improve complex sleep-related behaviours associated with rapid eye movement sleep behaviour disorder in Parkinson's disease patients: a case series. J Clin Pharm Ther 39:564–566. doi: 10.1111/jcpt.12179
