# Supplementary material for: A critical review of the pharmacological treatment of REM sleep behavior disorder in adults: time for more and larger randomized placebo-controlled trials
Source: J Neurol. 2021 Jan 7;269(1):125–48. doi: 10.1007/s00415-020-10353-0 (PMC8739295; doi:10.1007/s00415-020-10353-0)
Supplement: Supplementary file 2 — (PDF 173 KB) [file 415_2020_10353_MOESM2_ESM.docx]

**Supplementary Tables S1-S28:**  Number of clinical responders per drug type trialled to treat RBD in older adults to date.

**Title:** An update on the pharmacological treatment of REM sleep behavior disorder in adults: Time for more and larger randomized placebo-controlled trials.

**Authors**: Moran Gilat PhD, Nathaniel Marshall PhD, Dries Testelmans MD PhD, Bertien Buyse MD PhD, Simon JG Lewis MD PhD

**Corresponding author:**

Dr Moran Gilat PhD

Email: [moran.gilat@kuleuven.be](mailto:moran.gilat@kuleuven.be)

**Table of content**

| **Drug Class** | **Drug type** | **Table** | **Page** |
| --- | --- | --- | --- |
| Benzodiazepines | Clonazepam | **S1** | 3 |
|  | Clonazepam + Melatonin | **S2** | 6 |
|  | Clonazepam + Other add-on | **S3** | 7 |
|  | Temazepam | **S4** | 8 |
|  | Zopiclone | **S5** | 9 |
|  | Other | **S6** | 9 |
| Melatonin  (+ agonist) | Melatonin | **S7** | 10 |
|  | Melatonin +  Other add-on | **S8** | 11 |
|  | Ramelteon | **S9** | 12 |
|  | Agomelatine | **S10** | 12 |
| Dopamine  (+ agonist) | Levodopa | **S11** | 13 |
|  | Pramipexole | **S12** | 13 |
|  | Ropinirole | **S13** | 14 |
|  | Rotigotine | **S14** | 14 |
| Anticholinergic | Donepezil | **S15** | 15 |
|  | Rivastigmine | **S16** | 15 |
| Gabapentinoid | Gabapentin | **S17** | 16 |
|  | Pregabalin | **S18** | 16 |
| Noradrenergic agonist | Clonidine | **S19** | 17 |
|  |  |  |  |
|  |  |  |  |
|  |  |  |  |
|  |  |  |  |
|  |  |  |  |
|  |  |  |  |
|  |  |  |  |
| Antidepressants  (per class) | SSRI | **S20** | 17 |
|  | Tricyclic | **S21** | 18 |
|  | Other | **S22** | 19 |
| Antipsychotics | Mixed types | **S23** | 20 |
| Anticonvulants | Mixed types | **S24** | 20 |
| Gamma-hydroxybutyric acid | Sodium Oxybate | **S25** | 21 |
| Other | Yi-Gan San (Yokukansan) | **S26** | 22 |
|  | Cannabidiol | **S27** | 22 |
|  | Metropolol | **S28** | 23 |
|  | Aspirin | **S28** | 23 |
| **References** | | | 24 |
|  | | | |

Benzodiazepines

**Table S1: Reports on the clinical effectiveness of *Clonazepam* for treating RBD**

| **Study**  ***(First author, year)*** | **Ref** | **Study design** | **Clinical population** | **Total N** | **Treatment (mg/day)** | **RBD screening** | **Clinical effectiveness (N subjects)** | | |
| --- | --- | --- | --- | --- | --- | --- | --- | --- | --- |
|  |  |  |  |  |  |  | **YES** | **PARTIAL** | **NO** |
| Shin, 2019 | 1 | RCT | PD | 19 | Clonazepam (0.5) | PSG | 10 | 3 | 6 |
| Li, 2016 | 2 | POS | IRBD | 39 | Clonazepam (0.125-3) | PSG | 26 | 0 | 13 |
| Iranzo, 2005 | 3 | POS | IRBD | 39 | Clonazepam (0.25-CT) | PSG | 32 | 6 | 1 |
| Iranzo, 2005 | 3 | POS | PD | 45 | Clonazepam (0.25-CT) | PSG | 38 | 7 | 0 |
| Iranzo, 2005 | 3 | POS | MSA | 26 | Clonazepam (0.25-CT) | PSG | 20 | 6 | 0 |
| Lapierre, 1992 | 4 | POS | IRBD | 5 | Clonazepam (0.5 - 2) | PSG | 0 | 5 | 0 |
| Lee, 2020 | 5 | RMH | Mixed | 171 | Clonazepam (1 ± 0.5) | PSG | 147 | 0 | 24 |
| Abenza Abildúa, 2019 | 6 | RMH | Mixed* | 22 | Clonazepam (NR) | PSG | 19 | 0 | 3 |
| Fernandez-Acros, 2016 | 7 | RMH | Mixed | 183 | Clonazepam (0.25-4) | PSG | 92 (~) | 52 (~) | 39 |
| McCarter, 2013 | 8 | RMH | Mixed* | 18 | Clonazepam (<0.5-3) | PSG | 3 | 11 | 4 |
| Sasai, 2013 | 9 | RMH | IRBD | 17 | Clonazepam (0.6 ± 0.3, 0.25-2) | PSG | 15 | 0 | 2 |
| Anderson, 2009 | 10 | RMH | Mixed* | 36 | Clonazepam (0.25-4) | PSG | 15 | 0 | 21 |
| Bonakis, 2009A | 11 | RMH | Mixed | 26 | Clonazepam (NR) | PSG | 17 | 0 | 9 |
| Lin, 2009 | 12 | RMH | Mixed* | 44 | Clonazepam (0.5-2.0) | PSG | 0 | 41 | 3 |
| Wing, 2008 | 13 | RMH | Mixed* | 71 | Clonazepam (1.4 ± 1.4) | Clinical | 62 | 0 | 9 |
| Özekmekçi, 2005 | 14 | RMH | PD | 35 | Clonazepam (0.5-1) | Clinical | 35 | 0 | 0 |
| Boeve, 2003 | 15 | RMH | Mixed* | 8 | Clonazepam (0.5-1) | PSG | 0 | 0 | 8 |
| Olson, 2000 | 16 | RMH | Mixed | 38 | Clonazepam (0.25-1.5) | PSG | 21 | 12 | 5 |
| Boeve, 1998 | 17 | RMH | DLB | 11 | Clonazepam (NR) | PSG | 10 | 0 | 1 |
| Schenck, 2013, 1996^#^ | 18,  19 | RMH | Mixed | 27 | Clonazepam (NR) | PSG | 24 | 0 | 3 |
| Schenck, 1993 | 20 | RMH | Mixed*^%^ | 67 | Clonazepam (NR) | PSG | 53 | 8 | 6 |
| Schenck, 1991 | 21 | RMH | Mixed^$^ | 16 | Clonazepam (0.25-2.0) | Clinical, some had PSG | 12 | 2 | 2 |
| Moghadam, 2017 | 22 | CR | IRBD | 2 | Clonazepam (2) | PSG | 0 | 0 | 2 |
| Zhou, 2017 | 23 | CR | IRBD with childhood onset POD | 1 | Clonazepam (0.5) | PSG | 1 | 0 | 0 |
| Liebenthal, 2016 | 24 | CR | PD with DBS + mild OSA | 1 | Clonazepam (1) | PSG | 0 | 0 | 1 |
| Coelho, 2015 | 25 | CR | DLB+OSA | 1 | Clonazepam (NR) | PSG | 1 | 0 | 0 |
| Cosentino, 2014 | 26 | CR | Parkinsonism+NT1 | 1 | Clonazepam (0.5) | PSG | 1 | 0 | 0 |
| Weeks, 2014 | 27 | CR | IRBD | 1 | Clonazepam (1) | PSG | 1 | 0 | 0 |
| Nomura, 2013 | 28 | CR | PD | 1 | Clonazepam (1) | PSG | 0 | 0 | 1 |
| Di Giacopo, 2012 | 29 | CR | PD | 12 | Clonazepam (≤2) | PSG | 0 | 0 | 12 |
| Lo Coco, 2012 | 30 | CR | FTD | 1 | Clonazepam (1) | PSG | 1 | 0 | 0 |
| Shinno, 2010 | 31 | CR | Cancer Patients^¥^ | 3 | Clonazepam (0.5) | PSG | 3 | 0 | 0 |
| Bonakis, 2009B | 32 | CR | IRBD+PAPT | 1 | Clonazepam (0.75) | PSG | 0 | 1 | 0 |
| Shneerson, 2009 | 33 | CR | IRBD | 1 | Clonazepam (NR) | PSG | 0 | 0 | 1 |
| Xi, 2009 | 34 | CR | Pontine stroke | 1 | Clonazepam (0.25) | PSG | 1 | 0 | 0 |
| Oguri, 2008 | 35 | CR | IRBD+Parkinsonism | 1 | Clonazepam (NR) | PSG | 1 | 0 | 0 |
| Shinno, 2008 | 36 | CR | IRBD | 2 | Clonazepam (0.5-1) | PSG | 0 | 1 | 1 |
| Boeve, 2007 | 37 | CR | IRBD | 1 | Clonazepam (0.5) | PSG | 1 | 0 | 0 |
| Manni, 2005 | 38 | CR | IRBD | 2 | Clonazepam (0.5-1) | PSG | 2 | 0 | 0 |
| Thomas, 2004 | 39 | CR | IRBD | 1 | Clonazepam (0.5) | Clinical | 1 | 0 | 0 |
| Iranzo, 2003 | 40 | CR | Spinocere-bellar ataxia type 3* | 3 | Clonazepam (1) | PSG | 2 | 1 | 0 |
| Massironi, 2003 | 41 | CR | DLB | 3 | Clonazepam (0.3-0.5) | Clinical | 2 | 0 | 1 |
| Nash, 2003 | 42 | CR | IRBD | 1 | Clonazepam (NR) | PSG | 0 | 0 | 1 |
| Daly, 2002 | 43 | CR | IRBD | 2 | Clonazepam (1) | Clinical | 0 | 2 | 0 |
| Oksenberg, 2002 | 44 | CR | IRBD + PLMS and/or OSA | 6 | Clonazepam (0.5) | PSG | 4 | 0 | 2 |
| Kimura, 2000 | 45 | CR | Pontine ischemic lesion | 1 | Clonazepam (0.25) | PSG | 1 | 0 | 0 |
| Ringman, 2000 | 46 | CR | IRBD | 1 | Clonazepam (1.5) | PSG | 0 | 0 | 1 |
| Schuld, 1999 | 47 | CR | RBD + NT1 +PLMS | 1 | Clonazepam (0.5-1) | PSG | 0 | 0 | 1**!** |
| Chiu, 1997 | 48 | CR | IRBD | 2 | Clonazepam (0.75-1.25) | PSG | 1 | 1 | 0 |
| Morfis, 1997 | 49 | CR | IRBD | 1 | Clonazepam (0.5-1) | PSG | 1 | 0 | 0 |
| Uchiyama, 1995 | 50 | CR | IRBD | 1 | Clonazepam (0.5) | PSG | 1 | 0 | 0 |
| Schenck, 1987 | 51 | CR | Mixed | 5 | Clonazepam (0.5) | PSG | 5 | 0 | 0 |
| Schenck, 1986 | 52 | CR | IRBD | 2 | Clonazepam (0.5-1.5) | PSG | 2 | 0 | 0 |
| **Total clinical effectiveness for Clonazepam (N subjects)** | | | | | | | **684** | **159** | **183** |
| **Total clinical effectiveness for Clonazepam (% subjects)** | | | | | | | **66.7%** | **15.5%** | **17.8%** |

**NOTE: Scoring of effectiveness: YES = Responders - authors reported clear sustained benefits with no troublesome side-effects; PARTIAL = Partial responders - authors reported partial improvement with some RBD symptoms remaining or some non-troublesome adverse events; NO = Non-responders - patients and/or bed-partners reported no improvement or the drug had to be withdrawn or dosage changed due to troublesome side-effects. Mixed = Sample of interest consisted of a mixture of patients with a variety diagnoses besides RBD. Clinical = diagnosis of probable RBD based on clinical history or questionnaire data only; PSG = Polysomnography confirmed diagnosis of RBD. Dosage of treatment is presented as the range or mean ± SD. =One or more subjects could be <50 years of age; %=Sample includes six subjects with RBD + parasomnia overlap disorder and 3 subjects with a familial form of RBD with sleep walking, sleep terror, narcolepsy and periodic/aperiodic limb movements; #=Both papers describe the treatment outcomes on the same sample of RBD cases and are therefore listed together; $=Includes 1 RBD case with major depression disorder, 1 with a history of alcohol abuse, 1 with amphetamine drug abuse, 1 with chronic anxiety disorder, and 1 with recurrent major depression disorder and alcohol abuse whose parasomnia became worse during periods of alcohol abstinence. ¥ = Although RBD was confirmed with PSG, the RBD could have been of toxin-metabolic nature due to cancer treatment; !=Induced OSA; (~)=authors reported treatment was (partially) successful despite adverse events noted by some patients. Abbreviations: AD=Alzheimer’s disease; CR=Case report; CT=Until clinical resolution or tolerability; DLB=Dementia with lewy bodies; FTD=Frontotemporal dementia; IRBD=idiopathic REM sleep behavior disorder; MSA=Multiple system atrophy; NR=Not reported; NT1=Narcolepsy Type 1; OSA=Obstructive sleep apnea; PAPT=Palatal tremor with ataxia; PD= Parkinson’s disease; PLMS=Periodic limb movements; POD=Parasomnia overlap disorder; POS=Prospective open-label study; PSG=Polysomnography; RCT=Randomised Controlled Trial; Ref=Reference; RMH=Retrospective medical history.**

**Table S2: Reports on the clinical effectiveness of *Clonazepam + Melatonin* for reducing RBD**

| **Study**  ***(First author, year)*** | **Ref** | **Study design** | **Clinical population** | **Total N** | **Treatment (mg/day)** | **RBD screening** | **Clinical effectiveness (N subjects)** | | |
| --- | --- | --- | --- | --- | --- | --- | --- | --- | --- |
|  |  |  |  |  |  |  | **YES** | **PARTIAL** | **NO** |
| Fernandez-Acros, 2016 | 7 | RMH | Mixed | 24 | CZP (NR) + MLT (NR) | PSG | NR | NR | NR |
| Lee, 2020 | 5 | RMH | Mixed | 1 | CZP (NR) + MLT (NR) | PSG | 1 | 0 | 0 |
| McCarter, 2013 | 8 | RMH | Mixed* | 2 | CZP (NR) + MLT (NR) | PSG | 0 | 2 | 0 |
| Boeve, 2003 | 15 | RMH | Mixed* | 7 | CZP (0.5-1) + MLT (6-12) | PSG | 2 | 3 | 2 |
| Moghadam, 2017 | 22 | CR | IRBD | 1 | CZP (2) + MLT (5) | PSG | 0 | 0 | 1 |
| Pierre-Justin, 2017 | 53 | CR | Familial IRBD | 1 | CZP (2) + MLT (12) | PSG | 0 | 1 | 0 |
| Liebenthal, 2016 | 24 | CR | PD with DBS + mild OSA | 1 | CZP (1) + MLT (12) | PSG | 0 | 0 | 1 |
| **Total clinical effectiveness for Clonazepam + Melatonin (N subjects)** | | | | | | | **3** | **6** | **4** |
| **Total clinical effectiveness for Clonazepam + Melatonin (% subjects)** | | | | | | | **23.1%** | **46.1%** | **30.8%** |

***=One or more subjects could be <50 years of age. Abbreviations: CR=Case report; CZP=Clonazepam; DBS=Deep Brain Stimulation; IRBD=idiopathic REM sleep behavior disorder; MLT=Melatonin; NR=Not reported; OSA=Obstructive sleep apnea; PD= Parkinson’s disease; PSG=Polysomnography; Ref=Reference; RMH=Retrospective medical history.**

**Table S3: Reports on the clinical effectiveness of *Clonazepam + Other add-on therapies* for reducing RBD**

| **Study**  ***(First author, year)*** | **Ref** | **Study design** | **Clinical population** | **Total N** | **Treatment (mg/day)** | **RBD screening** | **Clinical effectiveness (N subjects)** | | |
| --- | --- | --- | --- | --- | --- | --- | --- | --- | --- |
|  |  |  |  |  |  |  | **YES** | **PARTIAL** | **NO** |
| Lee, 2020 | 5 | RMH | Mixed | 14 | CZP (NR) +  Carbamezapine (NR) | PSG | 6 | 0 | 8 |
| Lee, 2020 | 5 | RMH | Mixed | 3 | CZP (NR) + Zolpidem (NR) | PSG | 2 | 0 | 1 |
| Lee, 2020 | 5 | RMH | Mixed | 4 | CZP (NR) +  Carbamezapine (NR) + Zolpidem (NR) | PSG | 0 | 0 | 4 |
| Abenza Abildúa, 2019 | 6 | RMH | IRBD+ Insomnia | 1 | CZP (NR) +  Trazodone (NR) | PSG | 1 | 0 | 0 |
| Abenza Abildúa, 2019 | 6 | RMH | IRBD+PLMS | 2 | CZP (NR) +  Gabapentin (NR) | PSG | 2 | 0 | 0 |
| Sasai, 2013 | 9 | RMH | IRBD | 33 | CZP (0.7 ± 0.3) + Pramipexole (0.3 ± 0.1) | PSG | 25 | 0 | 8 |
| Anderson, 2009 | 10 | RMH | Mixed* | 1 | CZP (NR) + MLT (NR) + Gabapentin (NR) | PSG | 1 | 0 | 0 |
| Anderson, 2009 | 10 | RMH | Mixed* | 1 | CZP (NR) + Zoplicone (NR) | PSG | 1 | 0 | 0 |
| Moghadam, 2017 | 22 | CR | IRBD | 1 | CZP (2) +  Carbamazepine (400) | PSG | 0 | 0 | 1 |
| Moghadam, 2017 | 22 | CR | IRBD | 1 | CZP (2) + Lamotrigine (25) | PSG | 0 | 0 | 1 |
| Moghadam, 2017 | 22 | CR | IRBD | 1 | CZP (2) +  Pramipexole (0.36) | PSG | 0 | 0 | 1 |
| Moghadam, 2017 | 22 | CR | IRBD | 1 | CZP (2) +  Sodium Oxybate (3) | PSG | 1 | 0 | 0 |
| Liebenthal, 2016 | 24 | CR | PD with DBS + mild OSA | 1 | CZP (1) + MLT (12) + Ramelteon (NR) + Prazosin (NR) + Cyproheptadine (NR) | PSG | 0 | 0 | 1 |
| Yeh, 2010 | 54 | CR | AD | 1 | CZP (0.5) + Rivastigmine (4.5) | PSG | 1 | 0 | 0 |
| Shinno, 2008 | 36 | CR | IRBD | 2 | CZP (0.25-0.5) +  Yi-Gan San (7.5) | PSG | 1 | 1 | 0 |
| Chung, 1994 | 55 | CR | IRBD | 1 | CZP (0.75) +  Clomipramine (75) | PSG | 0 | 1 | 0 |
| Clarke, 2000 | 56 | CR | OSA + MD + MCI | 1 | CZP (0.75) + Setraline (150) | PSG | 0 | 1 | 0 |
| **Total clinical effectiveness for Clonazepam + Add-on therapies (N subjects)** | | | | | | | **41** | **3** | **25** |
| **Total clinical effectiveness for Clonazepam + Add-on therapies (% subjects)** | | | | | | | **59.4%** | **4.4%** | **36.2%** |

***=One or more subjects could be <50 years of age. Abbreviations: AD=Alzheimer’s Disease; CR=Case report; CZP=Clonazepam; DBS=Deep Brain Stimulation; IRBD=idiopathic REM sleep behavior disorder; MCI=Mild Cognitive Impairment; MLT=Melatonin; NR=Not reported; OSA=Obstructive sleep apnea; PD= Parkinson’s disease; PLMS=Periodic limb movements; PSG=Polysomnography; Ref=Reference; RMH=Retrospective medical history.**

**Table S4: Reports on the clinical effectiveness of *Temazepam* for reducing RBD**

| **Study**  ***(First author, year)*** | | **Ref** | **Study design** | **Clinical population** | **Total N** | **Treatment (mg/day)** | **RBD screening** | **Clinical effectiveness (N subjects)** | | |
| --- | --- | --- | --- | --- | --- | --- | --- | --- | --- | --- |
|  |  |  |  |  |  |  |  | **YES** | **PARTIAL** | **NO** |
| Anderson, 2009 | | 10 | RMH | Mixed* | 1 | Temazepam (NR) | PSG | 0 | 0 | 1 |
| Bonakis, 2009A | | 11 | RMH | IRBD | 1 | Temazepam (NR) | PSG | 1 | 0 | 0 |
| Shneerson, 2009 | | 33 | CR | IRBD + MD | 1 | Temazepam (NR) | PSG | 0 | 0 | 1 |
| **Total clinical effectiveness for Temazepam (N subjects)** | | | | | | | | **1** | **0** | **2** |
|  | **Total clinical effectiveness for Temazepam (% subjects)** | | | | | | | **33.3%** | **0%** | **66.7%** |

***=One or more subjects could be <50 years of age. Abbreviations: CR=Case report; IRBD=idiopathic REM sleep behavior disorder; MD=Major Depression; NR=Not reported; PSG=Polysomnography; Ref=Reference; RMH=Retrospective medical history.**

**Table S5: Reports on the clinical effectiveness of *Zopiclone* for reducing RBD**

| **Study**  ***(First author, year)*** | **Ref** | **Study design** | **Clinical population** | **Total N** | **Treatment (mg/day)** | **RBD screening** | **Clinical effectiveness (N subjects)** | | |
| --- | --- | --- | --- | --- | --- | --- | --- | --- | --- |
|  |  |  |  |  |  |  | **YES** | **PARTIAL** | **NO** |
| Anderson, 2009 | 10 | RMH | Mixed* | 9 | Zopiclone (3.75-7.5) | PSG | 6 | 0 | 3 |
| Bonakis, 2009A | 11 | RMH | IRBD | 1 | Zopiclone (NR) | PSG | 1 | 0 | 0 |
| Shneerson, 2009 | 33 | CR | IRBD+MD | 1 | Zopiclone (NR) | PSG | 0 | 0 | 1 |
| Nash, 2003 | 42 | CR | IRBD | 1 | Zopiclone (NR) | PSG | 0 | 0 | 1 |
| **Total clinical effectiveness for Zopiclone (N subjects)** | | | | | | | **7** | **0** | **5** |
| **Total clinical effectiveness for Zopiclone (% subjects)** | | | | | | | **58.3%** | **0%** | **41.7%** |

**NOTE: Zopiclone is a drug of the cyclopyrrolone class, which has similar GABAergic effects as benzodiazepines; *=One or more subjects could be <50 years of age. Abbreviations: CR=Case report; IRBD=idiopathic REM sleep behavior disorder; MD=Major Depression; NR=Not reported; PSG=Polysomnography; Ref=Reference; RMH=Retrospective medical history.**

**Table S6: Reports on the clinical effectiveness of *Other benzodiazepines* for reducing RBD**

| **Study**  ***(First author, year)*** | **Ref** | **Study design** | **Clinical population** | **Total N** | **Treatment (mg/day)** | **RBD screening** | **Clinical effectiveness (N subjects)** | | |
| --- | --- | --- | --- | --- | --- | --- | --- | --- | --- |
|  |  |  |  |  |  |  | **YES** | **PARTIAL** | **NO** |
| Escriba, 2016 | 57 | RMH | Mixed | 1 | ‘Benzodiazepines’ (0.5-2)^#^ | PSG | 1 | 0 | 0 |
| Anderson, 2009 | 10 | RMH | Mixed* | 1 | Temazepam + Zopiclone (NR) | PSG | 1 | 0 | 0 |
| Olson, 2000 | 16 | RMH | Mixed* | 2 | Triazolam (NR) | PSG | 1 | 0 | 1 |
| Fernandez-Acros, 2016 | 7 | RMH | Mixed | 14 | ‘Benzodiazepines’ (NR) | PSG | 0 | 0 | 14 |
| Shinno, 2008 | 36 | CR | IRBD | 1 | Nitrazepam (5) | PSG | 0 | 0 | 1 |
| Shinno, 2008 | 36 | CR | IRBD | 1 | Bromazepam (5) | PSG | 0 | 0 | 1 |
| Schenck, 1986 | 52 | CR | IRBD | 2 | Alprazolam (0.5) | PSG | 0 | 0 | 2 |
| **Total clinical effectiveness for Other benzodiazepines (N subjects)** | | | | | | | **3** | **0** | **19** |
| **Total clinical effectiveness for Other benzodiazepines (% subjects)** | | | | | | | **13.6%** | **0%** | **86.4%** |

**# - Authors do not report which benzodiazepines were administered; *=One or more subjects could be <50 years of age. Abbreviations: CR=Case report; IRBD=idiopathic REM sleep behavior disorder; NR=Not reported; PSG=Polysomnography; Ref=Reference; RMH=Retrospective medical history.**

Melatonin + agonist

**Table S7: Reports on the clinical effectiveness of *Melatonin* for reducing RBD**

| **Study**  ***(First author, year)*** | **Ref** | **Study design** | **Clinical population** | **Total N** | **Treatment (mg/day)** | **RBD screening** | **Clinical effectiveness (N subjects)** | | |
| --- | --- | --- | --- | --- | --- | --- | --- | --- | --- |
|  |  |  |  |  |  |  | **YES** | **PARTIAL** | **NO** |
| Gilat, 2020 | 58 | RCT | PD | 15 | PR-Melatonin (4) | PSG | 2 | 3 | 10 |
| Jun, 2019 | 59 | RCT | IRBD | 16 | PR-Melatonin (2-6) | PSG | 4 | 3 | 9 |
| Kunz, 2010 | 60 | RCT | Mixed* | 8 | Melatonin (3) | PSG | 7 | 1 | 0 |
| Takeuchi, 2001 | 61 | POS | NR | 15 | Melatonin (3-9) | PSG | 3 | 10 | 2 |
| Kunz, 1999 | 62 | POS | Mixed* | 6 | Melatonin (3) | PSG | 5 | 1 | 0 |
| Abenza Abildúa, 2019 | 6 | RMH | Mixed* | 7 | Melatonin (NR) | PSG | 3 | 0 | 4 |
| Escriba, 2016 | 57 | RMH | Mixed | 5 | PR-Melatonin (2) | PSG | 4 | 0 | 1 |
| Fernandez-Acros, 2016 | 7 | RMH | Mixed | 5 | Melatonin (1.9-9) | PSG | 0 | 1 | 4 |
| McCarter, 2013 | 8 | RMH | Mixed* | 25 | Melatonin (<6-25) | PSG | 3 | 15 | 7 |
| Anderson, 2009 | 10 | RMH | Mixed* | 2 | Melatonin (10) | PSG | 2 | 0 | 0 |
| Bonakis, 2009A | 11 | RMH | IRBD | 2 | Melatonin (NR) | PSG | 2 | 0 | 0 |
| Bonakis, 2009A | 11 | RMH | MSA | 1 | Melatonin (NR) | PSG | 1 | 0 | 0 |
| Boeve, 2003 | 15 | RMH | Mixed* | 9 | Melatonin (3-12) | PSG | 5 | 0 | 4 |
| Feemster, 2019 | 63 | CR | PTSD+OSA | 1 | Melatonin (3-6) | PSG | 0 | 0 | 1 |
| Xu, 2019 | 64 | CR | PD with childhood onset POD | 1 | Melatonin (3) | PSG | 1 | 0 | 0 |
| Kunz, 2018 | 65 | CR | PD | 1 | PR-Melatonin (2) | PSG | 1 | 0 | 0 |
| Pierre-Justin, 2017 | 53 | CR | Familial IRBD | 1 | Melatonin (6) | PSG | 1 | 0 | 0 |
| Wierzbicka, 2017 | 66 | CR | IRBD | 1 | Melatonin (5) | PSG | 0 | 1 | 0 |
| Felix, 2016 | 67 | CR | OSA + Pons cavernoma | 1 | Melatonin (NR) | PSG | 0 | 1 | 0 |
| Di Giacopo, 2012 | 29 | CR | PD | 12 | Melatonin (≤5) | PSG | 0 | 0 | 12 |
| Shneerson, 2009 | 33 | CR | IRBD + MD | 1 | Melatonin (NR) | PSG | 0 | 0 | 1 |
|  | 68 | CR | probable AD + OSA | 1 | Melatonin (10) | PSG | 0 | 1 | 0 |
| Kunz, 1997 | 69 | CR | IRBD | 1 | Melatonin (3) | PSG | 1 | 0 | 0 |
| **Total clinical effectiveness for Melatonin (N subjects)** | | | | | | | **45** | **37** | **55** |
| **Total clinical effectiveness for Melatonin (% subjects)** | | | | | | | **32.9%** | **27.0%** | **40.1%** |

***=One or more subjects could be <50 years of age. Abbreviations: AD=Alzheimer’s Disease; CR=Case report; CZP=Clonazepam; DBS=Deep Brain Stimulation; IRBD=idiopathic REM sleep behavior disorder; MCI=Mild Cognitive Impairment; MLT=Melatonin; NR=Not reported; OSA=Obstructive sleep apnea; PD= Parkinson’s disease; PLMS=Periodic limb movements; POD=Parasomnia Overlap Disoder; POS=Prospective open-label study; PR=Prolonged Release; PSG=Polysomnography; PTSD=Posttraumatic Stress Disorder; RCT=Randomised Controlled Trial; Ref=Reference; RMH=Retrospective medical history.**

**Table S8: Reports on the clinical effectiveness of *Melatonin + Add-on therapies* for reducing RBD**

| **Study**  ***(First author, year)*** | **Ref** | **Study design** | **Clinical population** | **Total N** | **Treatment (mg/day)** | **RBD screening** | **Clinical effectiveness (N subjects)** | | |
| --- | --- | --- | --- | --- | --- | --- | --- | --- | --- |
|  |  |  |  |  |  |  | **YES** | **PARTIAL** | **NO** |
| Abenza Abildúa, 2019 | 6 | RMH | IRBD | 1 | Melatonin +  Gabapentin (NR) | PSG | NR | NR | NR |
| Bonakis, 2009B | 32 | CR | IRBD+PAPT | 1 | Melatonin (3) +  Ropinorole (4) | PSG | 0 | 1 | 0 |
| Moghadam, 2017 | 22 | CR | IRBD | 1 | Melatonin (5) + Pramipexole (0.45) | PSG | 0 | 0 | 1 |
| Moghadam, 2017 | 22 | CR | IRBD | 1 | Melatonin (5) + Pramipexole (0.45) + Sodium Oxybate (4.5) | PSG | 0 | 1 | 0 |
| **Total clinical effectiveness for Melatonin + Add-on therapies (N subjects)** | | | | | | | **0** | **2** | **1** |
| **Total clinical effectiveness for Melatonin + Add-on therapies (% subjects)** | | | | | | | **0%** | **66.7%** | **33.3%** |

**Abbreviations: CR=Case report; IRBD=idiopathic REM sleep behavior disorder; NR=Not reported; PAPT=Palatal tremor with ataxia; PSG=Polysomnography; Ref=Reference; RMH=Retrospective medical history.**

**Table S9: Reports on the clinical effectiveness of *Ramelteon* for reducing RBD**

| **Study**  ***(First author, year)*** | **Ref** | **Study design** | **Clinical population** | **Total N** | **Treatment (mg/day)** | **RBD screening** | **Clinical effectiveness (N subjects)** | | |
| --- | --- | --- | --- | --- | --- | --- | --- | --- | --- |
|  |  |  |  |  |  |  | **YES** | **PARTIAL** | **NO** |
| Esaki, 2016 | 70 | POS | IRBD | 12 | Ramelteon (8) | PSG | 1 | 1 | 10 |
| Kashihara, 2016 | 71 | POS | PD | 24 | Ramelteon (8) | Clinical | NR | NR | NR |
| Kasanuki, 2013 | 72 | CR | DLB | 2 | Ramelteon (8) | Clinical | 2 | 0 | 0 |
| Nomura, 2013 | 28 | CR | MSA | 1 | Ramelteon (8) | PSG | 1 | 0 | 0 |
| Nomura, 2013 | 28 | CR | PD | 1 | Ramelteon (8) | PSG | 1 | 0 | 0 |
| **Total clinical effectiveness for Ramelteon (N subjects)** | | | | | | | **5** | **1** | **10** |
| **Total clinical effectiveness for Ramelteon (% subjects)** | | | | | | | **31.3%** | **6.2%** | **62.5%** |

**Abbreviations: CR=Case report; DLB=Dementia with lewy bodies; IRBD=idiopathic REM sleep behavior disorder; MSA=Multiple system atrophy; PD= Parkinson’s disease; POS=Prospective open-label study; PSG=Polysomnography; Ref=Reference.**

**Table S10: Reports on the clinical effectiveness of *Agomelatine* for reducing RBD**

| **Study**  ***(First author, year)*** | **Ref** | **Study design** | **Clinical population** | **Total N** | **Treatment (mg/day)** | **RBD screening** | **Clinical effectiveness (N subjects)** | | |
| --- | --- | --- | --- | --- | --- | --- | --- | --- | --- |
|  |  |  |  |  |  |  | **YES** | **PARTIAL** | **NO** |
| Bonakis, 2012 | 73 | CR | IRBD | 3 | Agomelatine (25-50) | PSG | 3 | 0 | 0 |
| **Total clinical effectiveness for Agomelatine (N subjects)** | | | | | | | **3** | **0** | **0** |
| **Total clinical effectiveness for Agomelatine (% subjects)** | | | | | | | **100%** | **0%** | **0%** |

**Abbreviations: CR=Case report; IRBD=idiopathic REM sleep behavior disorder; PSG=Polysomnography; Ref=Reference.**

Dopamine + agonist

**Table S11: Reports on the clinical effectiveness of *Levodopa* for reducing RBD**

| **Study**  ***(First author, year)*** | **Ref** | **Study design** | **Clinical population** | **Total N** | **Treatment (mg/day)** | **RBD screening** | **Clinical effectiveness (N subjects)** | | |
| --- | --- | --- | --- | --- | --- | --- | --- | --- | --- |
|  |  |  |  |  |  |  | **YES** | **PARTIAL** | **NO** |
| Fernandez-Acros, 2016 | 7 | RMH | Mixed | 1 | Levodopa (NR) | PSG | 0 | 0 | 1 |
| Bonakis, 2009A | 11 | RMH | IRBD | 3 | Levodopa (NR) | PSG | 3 | 0 | 0 |
| Bonakis, 2009A | 11 | RMH | PD | 3 | Levodopa (NR) | PSG | 3 | 0 | 0 |
| Özekmekçi, 2005 | 14 | RMH | PD | 10 | Levodopa (NR) | Clinical | 0 | 0 | 10 |
| Özekmekçi, 2005 | 14 | RMH | PD | 25 | Levodopa (NR) +  Dopamine agonist (NR) | Clinical | 0 | 0 | 25 |
| Tan, 1996 | 74 | CR | PD | 3 | Levodopa (NR) | Clinical | 2 | 1 | 0 |
| **Total clinical effectiveness for Levodopa (N subjects)** | | | | | | | **8** | **1** | **36** |
| **Total clinical effectiveness for Levodopa (% subjects)** | | | | | | | **17.8%** | **2.2%** | **80%** |

**Abbreviations: CR=Case report; IRBD=idiopathic REM sleep behavior disorder; NR= Not Reported; PD= Parkinson’s disease; PSG=Polysomnography; Ref=Reference; RMH=Retrospective medical history.**

**Table S12: Reports on the clinical effectiveness of *Pramipexole* for reducing RBD**

| **Study**  ***(First author, year)*** | **Ref** | **Study design** | **Clinical population** | **Total N** | **Treatment (mg/day)** | **RBD screening** | **Clinical effectiveness (N subjects)** | | |
| --- | --- | --- | --- | --- | --- | --- | --- | --- | --- |
|  |  |  |  |  |  |  | **YES** | **PARTIAL** | **NO** |
| Sasai, 2012 | 75 | POS | IRBD+PLMS | 15 | Pramipexole (0.21 ± 0.09, 0.125-1.5) | PSG | 12 | 0 | 3 |
| Kumru, 2008 | 76 | POS | PD | 11 | Pramipexole (0.54 - CT) | PSG | 0 | 0 | 11 |
| Fantini, 2003 | 77 | POS | IRBD | 8 | Pramipexole (0.5-1) | PSG | 5 | 0 | 3 |
| Fernandez-Acros, 2016 | 7 | RMH | Mixed | 1 | Pramipexole (NR) | PSG | 0 | 0 | 1 |
| Sasai, 2013 | 9 | RMH | IRBD | 81 | Pramipexole (0.2 ± 0.1, 0.125-1.5) | PSG | 50 | 0 | 31 |
| Schmidt, 2006 | 78 | CR | Mixed | 10 | Pramipexole (0.89 ± 0.31, 0.25-1.5) evening dose | PSG | 4 | 4 | 2 |
| **Total clinical effectiveness for Pramipexole (N subjects)** | | | | | | | **71** | **4** | **51** |
| **Total clinical effectiveness for Pramipexole (% subjects)** | | | | | | | **56.3%** | **3.2%** | **40.5%** |

**Abbreviations: CR=Case report; IRBD=idiopathic REM sleep behavior disorder; PD= Parkinson’s disease; PLMS=Periodic limb movements; POS=Prospective open-label study; PSG=Polysomnography; Ref=Reference; RMH=Retrospective medical history.**

**Table S13: Reports on the clinical effectiveness of *Ropinirole* for reducing RBD**

| **Study**  ***(First author, year)*** | **Ref** | **Study design** | **Clinical population** | **Total N** | **Treatment (mg/day)** | **RBD screening** | **Clinical effectiveness (N subjects)** | | |
| --- | --- | --- | --- | --- | --- | --- | --- | --- | --- |
|  |  |  |  |  |  |  | **YES** | **PARTIAL** | **NO** |
| Dušek, 2010 | 79 | POS | PD | 5 | PR-Ropinirole (17.2±6) | PSG | 0 | 0 | 5 |
| Fernandez-Acros, 2016 | 7 | RMH | Mixed | 1 | Ropinirole (NR) | PSG | 0 | 0 | 1 |
| Bonakis, 2009B^#^ | 32 | CR | IRBD+PAPT | 1 | Ropinirole (4) | PSG | 0 | 1 | 0 |
| **Total clinical effectiveness for Ropinirole (N subjects)** | | | | | | | **0** | **1** | **6** |
| **Total clinical effectiveness for Ropinirole (% subjects)** | | | | | | | **0%** | **14.3%** | **85.7%** |

**# = Presumably the same IRBD+PAPT patient is also reported by Bonakis 2009A. Abbreviations: CR=Case report; IRBD=idiopathic REM sleep behavior disorder; PAPT=Palatal tremor with ataxia; PD= Parkinson’s disease; POS=Prospective open-label study; PSG=Polysomnography; Ref=Reference.**

**Table S14: Reports on the clinical effectiveness of *Rotigotine* for reducing RBD**

| **Study**  ***(First author, year)*** | **Ref** | **Study design** | **Clinical population** | **Total N** | **Treatment (mg/day)** | **RBD screening** | **Clinical effectiveness (N subjects)** | | |
| --- | --- | --- | --- | --- | --- | --- | --- | --- | --- |
|  |  |  |  |  |  |  | **YES** | **PARTIAL** | **NO** |
| Wang, 2016 | 80 | POS | PD | 11 | Rotigotine (12.4 ± 4.3) | PSG | 7 | 0 | 4 |
| **Total clinical effectiveness for Rotigotine (N subjects)** | | | | | | | **0** | **1** | **5** |
| **Total clinical effectiveness for Rotigotine (% subjects)** | | | | | | | **63.6%** | **16.7%** | **36.4%** |

**Abbreviations: PD= Parkinson’s disease; POS=Prospective open-label study; PSG=Polysomnography; Ref=Reference.**

Anticholinergic

**Table 15: Reports on the clinical effectiveness of *Donepezil* for reducing RBD**

| **Study**  ***(First author, year)*** | **Ref** | **Study design** | **Clinical population** | **Total N** | **Treatment (mg/day)** | **RBD screening** | **Clinical effectiveness (N subjects)** | | |
| --- | --- | --- | --- | --- | --- | --- | --- | --- | --- |
|  |  |  |  |  |  |  | **YES** | **PARTIAL** | **NO** |
| Boeve, 2003 | 15 | RMH | DLB | 50 | Donepezil (NR) | NR | 0 | 0 | 50 |
| Ozaki, 2012 | 81 | CR | DLB | 1 | Donepezil (5) | PSG | 0 | 1 | 0 |
| Massironi, 2003 | 41 | CR | DLB | 3 | Donepezil (10) | Clinical | 1 | 0 | 2 |
| Ringman, 2000 | 46 | CR | IRBD | 1 | Donepezil (15) | PSG | 0 | 1 | 0 |
| Ringman, 2000 | 46 | CR | AD | 1 | Donepezil (10) | Clinical | 0 | 1 | 0 |
| **Total clinical effectiveness for Donepezil (N subjects)** | | | | | | | **1** | **3** | **52** |
| **Total clinical effectiveness for Donepezil (% subjects)** | | | | | | | **1.8%** | **5.4%** | **92.8%** |

**Abbreviations: AD=Alzheimer’s Disease; CR=Case report; DLB=Dementia with lewy bodies; IRBD=idiopathic REM sleep behavior disorder; NR=Not Reported; PSG=Polysomnography; Ref=Reference; RMH=Retrospective medical history.**

**Table S16: Reports on the clinical effectiveness of *Rivastigmine* for reducing RBD**

| **Study**  ***(First author, year)*** | **Ref** | **Study design** | **Clinical population** | **Total N** | **Treatment (mg/day)** | **RBD screening** | **Clinical effectiveness (N subjects)** | | |
| --- | --- | --- | --- | --- | --- | --- | --- | --- | --- |
|  |  |  |  |  |  |  | **YES** | **PARTIAL** | **NO** |
| Brunetti, 2014 | 82 | RCT | IRBD+MCI | 25 | Rivastigmine (4.6) | PSG | 18 | 0 | 7 |
| Di Giacopo, 2012 | 29 | RCT | PD | 10 | Rivastigmine (4.6) | PSG | 7 | 1 | 2 |
| Yeh, 2010 | 54 | CR | AD | 1 | Rivastigmine (4.5) | PSG | 0 | 0 | 1***** |
| **Total clinical effectiveness for Rivastigmine (N subjects)** | | | | | | | **25** | **1** | **10** |
| **Total clinical effectiveness for Rivastigmine (% subjects)** | | | | | | | **69.4%** | **2.8%** | **27.8%** |

***=Induced RBD. Abbreviations: AD=Alzheimer’s Disease; CR=Case report; IRBD=idiopathic REM sleep behavior disorder; MCI=Mild Cognitive Impairment; PD=Parkinson’s Disease; PSG=Polysomnography; RCT=Randomised Controlled Trial; Ref=Reference; RMH=Retrospective medical history.**

Gabapentinoid

**Table S17: Reports on the clinical effectiveness of *Gabapentin* for reducing RBD**

| **Study**  ***(First author, year)*** | **Ref** | **Study design** | **Clinical population** | **Total N** | **Treatment (mg/day)** | **RBD screening** | **Clinical effectiveness (N subjects)** | | |
| --- | --- | --- | --- | --- | --- | --- | --- | --- | --- |
|  |  |  |  |  |  |  | **YES** | **PARTIAL** | **NO** |
| Escriba, 2016 | 57 | RMH | Mixed | 14 | Gabapentin (300-800) | PSG | 12 | 0 | 2 |
| Anderson, 2009 | 10 | RMH | Mixed* | 1 | Gabapentin (NR) | PSG | 0 | 0 | 1 |
| Shneerson, 2009 | 33 | CR | IRBD+MD | 1 | Gabapentin (NR) | PSG | 0 | 0 | 1 |
| **Total clinical effectiveness for Gabapentin (N subjects)** | | | | | | | **12** | **0** | **4** |
| **Total clinical effectiveness for Gabapentin (% subjects)** | | | | | | | **75%** | **0%** | **25%** |

***=One or more subjects could be <50 years of age. Abbreviations: CR=Case report; IRBD=idiopathic REM sleep behavior disorder; MD=Major Depression; NR=Not Reported; PSG=Polysomnography; Ref=Reference; RMH=Retrospective medical history.**

**Table S18: Reports on the clinical effectiveness of *Pregabalin* for reducing RBD**

| **Study**  ***(First author, year)*** | **Ref** | **Study design** | **Clinical population** | **Total N** | **Treatment (mg/day)** | **RBD screening** | **Clinical effectiveness (N subjects)** | | |
| --- | --- | --- | --- | --- | --- | --- | --- | --- | --- |
|  |  |  |  |  |  |  | **YES** | **PARTIAL** | **NO** |
| Escriba, 2016 | 57 | RMH | Mixed | 3 | Pregabalin (75-150) | PSG | 2 | 0 | 1 |
| **Total clinical effectiveness for Pregabalin (N subjects)** | | | | | | | **2** | **0** | **1** |
| **Total clinical effectiveness for Pragabalin (% subjects)** | | | | | | | **66.7%** | **0%** | **33.3%** |

**Abbreviations: PSG=Polysomnography; Ref=Reference; RMH=Retrospective medical history.**

Noradrenergic agonist

**Table S19: Reports on the clinical effectiveness of *Clonidine* for reducing RBD**

| **Study**  ***(First author, year)*** | **Ref** | **Study design** | **Clinical population** | **Total N** | **Treatment (mg/day)** | **RBD screening** | **Clinical effectiveness (N subjects)** | | |
| --- | --- | --- | --- | --- | --- | --- | --- | --- | --- |
|  |  |  |  |  |  |  | **YES** | **PARTIAL** | **NO** |
| Shneerson, 2009 | 33 | CR | IRBD + MD | 1 | Clonidine (NR) | PSG | 0 | 0 | 1 |
| Nash, 2003 | 42 | CR | IRBD | 1 | Clonidine (100-200μg) | PSG | 1 | 0 | 0 |
| **Total clinical effectiveness for Clondine (N subjects)** | | | | | | | **1** | **0** | **1** |
| **Total clinical effectiveness for Clondine (% subjects)** | | | | | | | **50%** | **0%** | **50%** |

**Abbreviations: CR=Case report; IRBD=idiopathic REM sleep behavior disorder; MD=Major Depression; NR=Not Reported; PSG=Polysomnography; Ref=Reference.**

Antidepressants (per class)

**Table S20: Reports on the clinical effectiveness of *Selective Serotonin Reuptake Inhibitors* (*SSRI)* for reducing RBD**

| **Study**  ***(First author, year)*** | **Ref** | **Study design** | **Clinical population** | **Total N** | **Treatment (mg/day)** | **RBD screening** | **Clinical effectiveness (N subjects)** | | |
| --- | --- | --- | --- | --- | --- | --- | --- | --- | --- |
|  |  |  |  |  |  |  | **YES** | **PARTIAL** | **NO** |
| Yamamoto, 2006 | 83 | POS | IRBD | 19 | Paroxetine (10-40) | PSG | 0 | 16 | 3 |
| Shinno, 2008 | 36 | CR | IRBD | 1 | Paroxetine (20) | PSG | 0 | 0 | 1 |
| Takahashi, 2008 | 84 | CR | IRBD | 1 | Paroxetine (10) | PSG | 0 | 1 | 0 |
| Takahashi, 2008 | 84 | CR | IRBD | 1 | Fluvoxamine (50) | PSG | 0 | 0 | 1 |
| Nash, 2003 | 42 | CR | IRBD | 1 | Trazodone (NR) | PSG | 0 | 0 | 1 |
| Clarke, 2000 | 56 | CR | OSA + MD + MCI | 1 | Setraline (100-150) | PSG | 0 | 0 | 1 |
| **Total clinical effectiveness for SSRI (N subjects)** | | | | | | | **0** | **17** | **7** |
| **Total clinical effectiveness for SSRI (% subjects)** | | | | | | | **0%** | **70.8%** | **29.2%** |

**Abbreviations: CR=Case report; IRBD=idiopathic REM sleep behavior disorder; MCI=Mild Cognitive Impairment; NR=Not reported; OSA=Obstructive sleep apnea; POS=Prospective open-label study; PSG=Polysomnography; Ref=Reference.**

**Table S21: Reports on the clinical effectiveness of *Tryciclic antidepressants* for reducing RBD**

| **Study**  ***(First author, year)*** | **Ref** | **Study design** | **Clinical population** | **Total N** | **Treatment (mg/day)** | **RBD screening** | **Clinical effectiveness (N subjects)** | | |
| --- | --- | --- | --- | --- | --- | --- | --- | --- | --- |
|  |  |  |  |  |  |  | **YES** | **PARTIAL** | **NO** |
| Fernandez-Acros, 2016 | 7 | RMH | Mixed | 1 | Imipramine (NR) | PSG | 0 | 0 | 1 |
| Fernandez-Acros, 2016 | 7 | RMH | Mixed | 1 | Carbamazepine (NR) | PSG | 0 | 0 | 1 |
| Nash, 2003 | 42 | CR | IRBD | 1 | Amytriptyline (NR) | PSG | 0 | 0 | 1 |
| Clarke, 2000 | 56 | CR | OSA + MD  + MCI | 1 | Dothiepin (150) | PSG | 0 | 0 | 1 |
| Chung, 1994 | 55 | CR | IRBD | 1 | Clomipramine (100) | PSG | 0 | 0 | 1 |
| Bamford, 1993 | 85 | CR | IRBD | 1 | Carbamazepine (100) | PSG | 1 | 0 | 0 |
| Schenck, 1986 | 52 | CR | IRBD | 1 | Amitriptyline (50) | PSG | 0 | 0 | 1 |
| Schenck, 1986 | 52 | CR | IRBD | 2 | Desipramine (50-250) | PSG | 0 | 0 | 2 |
| **Total clinical effectiveness for Tricyclic antipsychotics (N subjects)** | | | | | | | **1** | **0** | **8** |
| **Total clinical effectiveness for Tricyclic antipsychotics (% subjects)** | | | | | | | **11.1%** | **0%** | **88.9%** |

**Abbreviations: CR=Case report; IRBD=idiopathic REM sleep behavior disorder; MCI=Mild Cognitive Impairment; NR=Not reported; OSA=Obstructive sleep apnea; PSG=Polysomnography; Ref=Reference.**

**Table S22: Reports on the clinical effectiveness of *Other antidepressants* for reducing RBD**

| **Study**  ***(First author, year)*** | **Ref** | **Study design** | **Clinical population** | **Total N** | **Treatment (mg/day)**  **-*Type of antidepressant*** | **RBD screening** | **Clinical effectiveness (N subjects)** | | |
| --- | --- | --- | --- | --- | --- | --- | --- | --- | --- |
|  |  |  |  |  |  |  | **YES** | **PARTIAL** | **NO** |
| Takahashi, 2008 | 84 | CR | IRBD | 1 | Mianserin (10)  -*Tetracyclic* | PSG | 0 | 0 | 1 |
| Takahashi, 2008 | 84 | CR | IRBD | 1 | Tandospirone (NR)  -*Azapirone* | PSG | 0 | 0 | 1***** |
| Nash, 2003 | 42 | CR | IRBD | 1 | Nefazodone (NR)  -SARI | PSG | 0 | 0 | 1 |
| Nash, 2003 | 42 | CR | IRBD | 1 | Venlafaxine (NR)  -*SNDRI* | PSG | 0 | 0 | 1 |
| Onofrj, 2003 | 86 | CR | PD | 4 | Mirtazapine (15-30)  -*NaSSA* | PSG | 0 | 0 | 4****** |
| **Total clinical effectiveness for Other types of antipsychotics (N subjects)** | | | | | | | **0** | **0** | **8** |
| **Total clinical effectiveness for Other types of antipsychotics (% subjects)** | | | | | | | **0%** | **0%** | **100%** |

**# = Although RBD was confirmed with PSG, it could have been of toxin-metabolic nature due to cancer treatment. Abbreviations: CR=Case report; IRBD=idiopathic REM sleep behavior disorder; NaSSA=Noradrenergic and specific serotonergic antipsychotic; NR=Not reported; PD=Parkinson’s Disease; PSG=Polysomnography; Ref=Reference; SARI=Serotonin antagonist and reuptake inhibitor; SNDRI=Serotonin-norepinephrine-dopamine reuptake inhibitor. *=Worsened RBD; ** =Induced RBD.**

**Table S23: Reports on the clinical effectiveness of A*ntipsychotics* for reducing RBD**

| **Study**  ***(First author, year)*** | **Ref** | **Study design** | **Clinical population** | **Total N** | **Treatment (mg/day)** | **RBD screening** | **Clinical effectiveness (N subjects)** | | |
| --- | --- | --- | --- | --- | --- | --- | --- | --- | --- |
|  |  |  |  |  |  |  | **YES** | **PARTIAL** | **NO** |
| Lin, 2009 | 12 | RMH | NR | 1 | Quetiapine (25) | PSG | 1 | 0 | 0 |
| Olson, 2000 | 16 | RMH | RBD + dementia | 2 | Clozapine (NR) | PSG | 1 | 1 | 0 |
| Boeve 1998 | 17 | RMH | DLB | 1 | Clozapine (NR) | PSG | 1 | 0 | 0 |
| Fernandez-Acros, 2016 | 7 | RMH | Mixed | 1 | Haloperidol (NR) | PSG | 0 | 0 | 1 |
| Shinno, 2010 | 31 | CR | Cancer patients^#^ | 1 | Haloperidol (5)  *-Buryrophenone* | PSG | 0 | 0 | 1 |
| Shinno, 2010 | 31 | CR | Cancer patients^#^ | 2 | Quetiapine (25) | PSG | 0 | 0 | 2 |
| Schenck, 1987 | 51 | CR | RBD + dementia | 1 | Haloperidol (NR)  *-Buryrophenone* | PSG | 0 | 0 | 1 |
| **Total clinical effectiveness for Atypical antipsychotics (N subjects)** | | | | | | | **3** | **1** | **5** |
| **Total clinical effectiveness for Atypical antipsychotics (% subjects)** | | | | | | | **33.3%** | **11.1%** | **55.6%** |

**# = Although RBD was confirmed with PSG, it could have been of toxin-metabolic nature due to cancer treatment. Abbreviations: CR=Case report; DLB=Dementia with Lewy Bodies; NR=Not reported; PSG=Polysomnography; RBD= REM sleep behavior disorder; Ref=Reference; RMH=Retrospective medical history.**

**Table S24: Reports on the clinical effectiveness of *Anticonvulants* for reducing RBD**

| **Study**  ***(First author, year)*** | **Ref** | **Study design** | **Clinical population** | **Total N** | **Treatment (mg/day)** | **RBD screening** | **Clinical effectiveness (N subjects)** | | |
| --- | --- | --- | --- | --- | --- | --- | --- | --- | --- |
|  |  |  |  |  |  |  | **YES** | **PARTIAL** | **NO** |
| Fernandez-Acros, 2016 | 7 | RMH | Mixed | 1 | Phenobarbital (NR) | PSG | 0 | 0 | 1 |
| Fernandez-Acros, 2016 | 7 | RMH | Mixed | 1 | Lamotrigine (NR) | PSG | 0 | 0 | 1 |
| Fernandez-Acros, 2016 | 7 | RMH | Mixed | 1 | Oxcarbazepine (NR) | PSG | 0 | 0 | 1 |
| **Total clinical effectiveness for Gabapentin (N subjects)** | | | | | | | **0** | **0** | **3** |
| **Total clinical effectiveness for Gabapentin (% subjects)** | | | | | | | **0%** | **0%** | **100%** |

**Abbreviations: NR=Not Reported; PSG=Polysomnography; Ref=Reference; RMH=Retrospective medical history.**

Gamma-hydroxybutyric acid (GHB)

**Table S25: Reports on the clinical effectiveness of *Sodium Oxybate* for reducing RBD**

| **Study**  ***(First author, year)*** | **Ref** | **Study design** | **Clinical population** | **Total N** | **Treatment (mg/day)** | **RBD screening** | **Clinical effectiveness (N subjects)** | | |
| --- | --- | --- | --- | --- | --- | --- | --- | --- | --- |
|  |  |  |  |  |  |  | **YES** | **PARTIAL** | **NO** |
| Anderson, 2009 | 10 | RMH | Mixed* | 1 | Sodium Oxybate (NR) | PSG | 1 | 0 | 0 |
| Moghadam, 2017 | 22 | CR | IRBD | 1 | Sodium oxybate (4.5) +  Pramipexole (0.45) | PSG | 1 | 0 | 0 |
| Liebenthal, 2016 | 24 | CR | PD with DBS + mild OSA | 1 | Sodium Oxybate (5.5) | PSG | 1 | 0 | 0 |
| Mayer, 2016 | 87 | CR | NT1 | 1 | Sodium Oxybate (6) | PSG | 1 | 0 | 0 |
| Shneerson, 2009 | 33 | CR | IRBD + MD | 1 | Sodium Oxybate (4.5) | PSG | 1 | 0 | 0 |
| **Total clinical effectiveness for Sodium Oxybate (N subjects)** | | | | | | | **5** | **0** | **0** |
| **Total clinical effectiveness for Sodium Oxybate (% subjects)** | | | | | | | **100%** | **0%** | **0%** |

***=One or more subjects could be <50 years of age. Abbreviations: CR=Case report; DBS=Deep Brain Stimulation; IRBD=idiopathic REM sleep behavior disorder; MD=Major Depression; NR=Not reported; NT1=Narcolepsy Type 1; OSA=Obstructive sleep apnea; PSG=Polysomnography; Ref=Reference.**

Other drugs or combination of drugs trialled for reducing RBD

**Table S26: Reports on the clinical effectiveness of *Yi-Gan San (Yokukansan)* for reducing RBD**

| **Study**  ***(First author, year)*** | **Ref** | **Study design** | **Clinical population** | **Total N** | **Treatment (mg/day)** | **RBD screening** | **Clinical effectiveness (N subjects)** | | |
| --- | --- | --- | --- | --- | --- | --- | --- | --- | --- |
|  |  |  |  |  |  |  | **YES** | **PARTIAL** | **NO** |
| Matsui, 2019 | 88 | RMH | IRBD | 17 | Yi-Gan San (2.5-5) | PSG | 12 | 0 | 5 |
| Matsui, 2019 | 88 | RMH | IRBD | 19 | Yi-Gan San (2.5-5) + Add-on clonazepam (NR) and/or pramipexole (NR) | PSG | 4 | 0 | 15 |
| Shinno, 2008 | 36 | CR | IRBD | 1 | Yi-Gan San (2.5) | PSG | 1 | 0 | 0 |
| **Total clinical effectiveness for Yi-Gan San (N subjects)** | | | | | | | **17** | **0** | **20** |
| **Total clinical effectiveness for Yi-Gan San (% subjects)** | | | | | | | **45.9%** | **0%** | **54.1%** |

**Abbreviations: CR=Case report; IRBD=idiopathic REM sleep behavior disorder; NR=Not reported; POS=Prospective open-label study; PSG=Polysomnography; Ref=Reference.**

**Table S27: Reports on the clinical effectiveness of *Cannabidiol* for reducing RBD**

| **Study**  ***(First author, year)*** | **Ref** | **Study design** | **Clinical population** | **Total N** | **Treatment (mg/day)** | **RBD screening** | **Clinical effectiveness (N subjects)** | | |
| --- | --- | --- | --- | --- | --- | --- | --- | --- | --- |
|  |  |  |  |  |  |  | **YES** | **PARTIAL** | **NO** |
| Chagas, 2014 | 89 | CR | PD | 4 | Cannabidiol (75-300) | 2 PSG, 2 Clinical | 4 | 0 | 0 |
| **Total clinical effectiveness for Cannabidiol (N subjects)** | | | | | | | **4** | **0** | **0** |
| **Total clinical effectiveness for Cannabidiol (% subjects)** | | | | | | | **100%** | **0%** | **0%** |

**Abbreviations: CR=Case report; PD=Parkinson’s disease; PSG=Polysomnography; Ref=Reference.**

**Table S28: Reports on the clinical effectiveness of *cardiac medications* for reducing RBD**

| **Study**  ***(First author, year)*** | **Ref** | **Study design** | **Clinical population** | **Total N** | **Treatment (mg/day)** | **RBD screening** | **Clinical effectiveness (N subjects)** | | |
| --- | --- | --- | --- | --- | --- | --- | --- | --- | --- |
|  |  |  |  |  |  |  | **YES** | **PARTIAL** | **NO** |
| Schenck, 1987 | 51 | CR | IRBD | 1 | Metoprolol (NR) + Aspirin (NR) | PSG | 0 | 0 | 1 |
| Schenck, 1987 | 51 | CR | IRBD | 1 | Aspirin (NR) | PSG | 0 | 0 | 1 |
| **Total clinical effectiveness for Metropolol (N subjects)** | | | | | | | **0** | **0** | **2** |
| **Total clinical effectiveness for Metropolol (% subjects)** | | | | | | | **0%** | **0%** | **100%** |

**NOTE: Authors of this study reported that a physician other than the authors themselves unsuccessfully tried to treat RBD with metoprolol and aspirin. Abbreviations: CR=Case report; IRBD=idiopathic REM sleep behavior disorder; NR=Not reported; PSG=Polysomnography; Ref=Reference.**

**References:**

1. Shin C, Park H, Lee W-W, et al (2019) Clonazepam for probable REM sleep behavior disorder in Parkinson's disease: A randomized placebo-controlled trial. Journal of the Neurological Sciences 401:81–86. doi: 10.1016/j.jns.2019.04.029

2. Li SX, Lam SP, Zhang J, et al (2016) A prospective, naturalistic follow-up study of treatment outcomes with clonazepam in rapid eye movement sleep behavior disorder. Sleep Med 21:114–120. doi: 10.1016/j.sleep.2015.12.020

3. Iranzo A, Santamaria J, Rye DB, et al (2005) Characteristics of idiopathic REM sleep behavior disorder and that associated with MSA and PD. Neurology 65:247–252. doi: 10.1212/01.wnl.0000168864.97813.e0

4. Lapierre O, Montplaisir J (1992) Polysomnographic features of REM sleep behavior disorder: development of a scoring method. Neurology 42:1371–1374. doi: 10.1212/wnl.42.7.1371

5. Lee HJ, Choi H, Yoon I-Y (2020) Age of Diagnosis and Comorbid PLMD Predict Poor Response of REM Behavior Disorder to Clonazepam. J Geriatr Psychiatry Neurol 9:891988720915517. doi: 10.1177/0891988720915517

6. Abenza Abildúa MJ, Miralles Martinez A, Arpa Gutiérrez FJ, et al (2019) Conditions associated with REM sleep behaviour disorder: Description of a hospital series. Neurologia 34:159–164. doi: 10.1016/j.nrl.2016.11.011

7. Fernández-Arcos A, Iranzo A, Serradell M, et al (2016) The Clinical Phenotype of Idiopathic Rapid Eye Movement Sleep Behavior Disorder at Presentation: A Study in 203 Consecutive Patients. Sleep 39:121–132. doi: 10.5665/sleep.5332

8. McCarter SJ, Boswell CL, St Louis EK, et al (2013) Treatment outcomes in REM sleep behavior disorder. Sleep Med 14:237–242. doi: 10.1016/j.sleep.2012.09.018

9. Sasai T, Matsuura M, Inoue Y (2013) Factors associated with the effect of pramipexole on symptoms of idiopathic REM sleep behavior disorder. Parkinsonism and Related Disorders 19:153–157. doi: 10.1016/j.parkreldis.2012.08.010

10. Anderson KN, Shneerson JM (2009) Drug treatment of REM sleep behavior disorder: the use of drug therapies other than clonazepam. J Clin Sleep Med 5:235–239.

11. Bonakis A, Howard RS, Ebrahim IO, et al (2009) REM sleep behaviour disorder (RBD) and its associations in young patients. Sleep Med 10:641–645. doi: 10.1016/j.sleep.2008.07.008

12. Lin F-C, Lai C-L, Huang P, et al (2009) The rapid-eye-movement sleep behavior disorder in Chinese-Taiwanese patients. Psychiatry Clin Neurosci 63:557–562. doi: 10.1111/j.1440-1819.2009.01998.x

13. Wing YK, Lam SP, Li SX, et al (2008) REM sleep behaviour disorder in Hong Kong Chinese: clinical outcome and gender comparison. J Neurol Neurosurg Psychiatr 79:1415–1416. doi: 10.1136/jnnp.2008.155374

14. Ozekmekçi S, Apaydin H, Kiliç E (2005) Clinical features of 35 patients with Parkinson's disease displaying REM behavior disorder. Clin Neurol Neurosurg 107:306–309. doi: 10.1016/j.clineuro.2004.09.021

15. Boeve BF, Silber MH, Ferman TJ (2003) Melatonin for treatment of REM sleep behavior disorder in neurologic disorders: results in 14 patients. Sleep Med 4:281–284.

16. Olson EJ, Boeve BF, Silber MH (2000) Rapid eye movement sleep behaviour disorder: demographic, clinical and laboratory findings in 93 cases. Brain 123 ( Pt 2):331–339. doi: 10.1093/brain/123.2.331

17. Boeve BF, Silber MH, Ferman TJ, et al (1998) REM sleep behavior disorder and degenerative dementia: an association likely reflecting Lewy body disease. Neurology 51:363–370. doi: 10.1212/wnl.51.2.363

18. Schenck CH, Boeve BF, Mahowald MW (2013) Delayed emergence of a parkinsonian disorder or dementia in 81% of older men initially diagnosed with idiopathic rapid eye movement sleep behavior disorder: a 16-year update on a previously reported series. Sleep Med 14:744–748. doi: 10.1016/j.sleep.2012.10.009

19. Schenck CH, Bundlie SR, Mahowald MW (1996) Delayed emergence of a parkinsonian disorder in 38% of 29 older men initially diagnosed with idiopathic rapid eye movement sleep behaviour disorder. Neurology 46:388–393. doi: 10.1212/wnl.46.2.388

20. Schenck C, Hurwitz T, Mahowald M (1993) Symposium: Normal and abnormal REM sleep regulation: REM sleep behaviour disorder: an update on a series of 96 patients and a review of the world literature. J Sleep Res 2:224–231. doi: 10.1111/j.1365-2869.1993.tb00093.x

21. Schenck CH, Mahowald MW (1991) Injurious sleep behavior disorders (parasomnias) affecting patients on intensive care units. Intensive Care Med 17:219–224. doi: 10.1007/BF01709881

22. Moghadam KK, Pizza F, Primavera A, et al (2017) Sodium oxybate for idiopathic REM sleep behavior disorder: a report on two patients. Sleep Med 32:16–21. doi: 10.1016/j.sleep.2016.04.014

23. Zhou J, Liang B, Du L, et al (2017) A patient with childhood-onset aggressive parasomnia diagnosed 50 years later with idiopathic REM sleep behavior disorder and a history of sleepwalking. Clin Neurol Neurosurg 160:105–107. doi: 10.1016/j.clineuro.2017.07.001

24. Liebenthal J, Valerio J, Ruoff C, Mahowald M (2016) A Case of Rapid Eye Movement Sleep Behavior Disorder in Parkinson Disease Treated With Sodium Oxybate. JAMA Neurol 73:126–127. doi: 10.1001/jamaneurol.2015.2904

25. Coelho FMS, Kim LJ, Cremaschi RC, et al (2015) Continuous positive airway pressure treatment associated with face injury during rapid eye movement behavior disorder. Sleep Med 16:805–806. doi: 10.1016/j.sleep.2014.11.021

26. Cosentino FII, Distefano A, Plazzi G, et al (2014) A case of REM sleep behavior disorder, narcolepsy-cataplexy, parkinsonism, and rheumatoid arthritis. Behav Neurol 2014:572931–6. doi: 10.1155/2014/572931

27. Weeks RG, D'Costa JA, Aiyappan V, et al (2014) Rapid eye movement behaviour disorder. Med J Aust 200:487–488. doi: 10.5694/mja13.11000

28. Nomura T, Kawase S, Watanabe Y, Nakashima K (2013) Use of ramelteon for the treatment of secondary REM sleep behavior disorder. Intern Med 52:2123–2126. doi: 10.2169/internalmedicine.52.9179

29. Di Giacopo R, Fasano A, Quaranta D, et al (2012) Rivastigmine as alternative treatment for refractory REM behavior disorder in Parkinson's disease. Mov Disord 27:559–561. doi: 10.1002/mds.24909

30. Coco Lo D, Cupidi C, Mattaliano A, et al (2012) REM sleep behavior disorder in a patient with frontotemporal dementia. Neurol Sci 33:371–373. doi: 10.1007/s10072-011-0702-5

31. Shinno H, Kamei M, Maegawa T, et al (2010) Three patients with cancer who developed rapid-eye-movement sleep behavior disorder. J Pain Symptom Manage 40:449–452. doi: 10.1016/j.jpainsymman.2010.01.016

32. Bonakis A, Papageorgiou SG, Merritt S, Williams AJ (2009) REM behaviour disorder preceding palatal tremor. Sleep Med 10:1161–1163. doi: 10.1016/j.sleep.2009.04.007

33. Shneerson JM (2009) Successful treatment of REM sleep behavior disorder with sodium oxybate. Clin Neuropharmacol 32:158–159. doi: 10.1097/WNF.0b013e318193e394

34. Xi Z, Luning W (2009) REM sleep behavior disorder in a patient with pontine stroke. Sleep Med 10:143–146. doi: 10.1016/j.sleep.2007.12.002

35. Oguri T, Tachibana N, Mitake S, et al (2008) Decrease in myocardial 123I-MIBG radioactivity in REM sleep behavior disorder: two patients with different clinical progression. Sleep Med 9:583–585. doi: 10.1016/j.sleep.2007.08.006

36. Shinno H, Kamei M, Nakamura Y, et al (2008) Successful treatment with Yi-Gan San for rapid eye movement sleep behavior disorder. Prog Neuropsychopharmacol Biol Psychiatry 32:1749–1751. doi: 10.1016/j.pnpbp.2008.06.015

37. Boeve BF, Dickson DW, Olson EJ, et al (2007) Insights into REM sleep behavior disorder pathophysiology in brainstem-predominant Lewy body disease. Sleep Med 8:60–64. doi: 10.1016/j.sleep.2006.08.017

38. Manni R, Terzaghi M (2005) REM behavior disorder associated with epileptic seizures. Neurology 64:883–884. doi: 10.1212/01.WNL.0000152894.47873.53

39. Thomas M, Moore K (2004) Falling asleep. Age Ageing 33:636–637. doi: 10.1093/ageing/afh180

40. Iranzo A, Muñoz E, Santamaria J, et al (2003) REM sleep behavior disorder and vocal cord paralysis in Machado-Joseph disease. Mov Disord 18:1179–1183. doi: 10.1002/mds.10509

41. Massironi G, Galluzzi S, Frisoni GB (2003) Drug treatment of REM sleep behavior disorders in dementia with Lewy bodies. Int Psychogeriatr 15:377–383. doi: 10.1017/s1041610203009621

42. Nash JR, Wilson SJ, Potokar JP, Nutt DJ (2003) Mirtazapine induces REM sleep behavior disorder (RBD) in parkinsonism. Neurology 61:1161–author reply 1161. doi: 10.1212/wnl.61.8.1161

43. Daly JJ, Compton SA (2002) Rapid eye movement (REM) sleep behaviour disorder; an easily missed diagnosis, a readily treatable condition. Ulster Med J 71:62–65.

44. Oksenberg A, Radwan H, Arons E, et al (2002) Rapid Eye Movement (REM) sleep behavior disorder: a sleep disturbance affecting mainly older men. Isr J Psychiatry Relat Sci 39:28–35.

45. Kimura K, Tachibana N, Kohyama J, et al (2000) A discrete pontine ischemic lesion could cause REM sleep behavior disorder. Neurology 55:894–895. doi: 10.1212/wnl.55.6.894

46. Ringman JM, Simmons JH (2000) Treatment of REM sleep behavior disorder with donepezil: a report of three cases. Neurology 55:870–871. doi: 10.1212/wnl.55.6.870

47. Schuld A, Kraus T, Haack M, et al (1999) Obstructive sleep apnea syndrome induced by clonazepam in a narcoleptic patient with REM-sleep-behavior disorder. J Sleep Res 8:321–322. doi: 10.1046/j.1365-2869.1999.00162.x

48. Chiu HF, Wing YK, Chung DW, Ho CK (1997) REM sleep behaviour disorder in the elderly. Int J Geriatr Psychiatry 12:888–891.

49. Morfis L, Schwartz RS, Cistulli PA (1997) REM sleep behaviour disorder: a treatable cause of falls in elderly people. Age Ageing 26:43–44. doi: 10.1093/ageing/26.1.43

50. Uchiyama M, Isse K, Tanaka K, et al (1995) Incidental Lewy body disease in a patient with REM sleep behavior disorder. Neurology 45:709–712. doi: 10.1212/wnl.45.4.709

51. Schenck CH, Bundlie SR, Patterson AL, Mahowald MW (1987) Rapid eye movement sleep behavior disorder. A treatable parasomnia affecting older adults. JAMA 257:1786–1789.

52. Schenck CH, Bundlie SR, Ettinger MG, Mahowald MW (1986) Chronic behavioral disorders of human REM sleep: a new category of parasomnia. Sleep 9:293–308. doi: 10.1093/sleep/9.2.293

53. Pierre-Justin A, Lannuzel A, Arnulf I (2017) Familial idiopathic rapid eye movement sleep behavior disorder. Sleep Med 30:29–30. doi: 10.1016/j.sleep.2016.04.015

54. Yeh S-B, Yeh P-Y, Schenck CH (2010) Rivastigmine-induced REM sleep behavior disorder (RBD) in a 88-year-old man with Alzheimer's disease. J Clin Sleep Med 6:192–195.

55. Chung KF, Wong MT (1994) Rapid eye movement sleep behaviour disorder in a Chinese male. Aust N Z J Psychiatry 28:144–146. doi: 10.3109/00048679409075857

56. Clarke NA, Williams AJ, Kopelman MD (2000) Rapid eye movement sleep behaviour disorder, depression and cognitive impairment. Case study. Br J Psychiatry 176:189–192. doi: 10.1192/bjp.176.2.189

57. Escribá J, Hoyo B (2016) Alternatives to Clonazepam in REM Behavior Disorder Treatment. J Clin Sleep Med 12:1193–1193. doi: 10.5664/jcsm.6068

58. Gilat M, Coeytaux Jackson A, Marshall NS, et al (2020) Melatonin for rapid eye movement sleep behavior disorder in Parkinson's disease: A randomised controlled trial. Mov Disord 35:344–349. doi: 10.1002/mds.27886

59. Jun J-S, Kim R, Byun J-I, et al (2019) Prolonged-release melatonin in patients with idiopathic REM sleep behavior disorder. Ann Clin Transl Neurol 6:716–722. doi: 10.1002/acn3.753

60. Kunz D, Mahlberg R (2010) A two-part, double-blind, placebo-controlled trial of exogenous melatonin in REM sleep behaviour disorder. J Sleep Res 19:591–596. doi: 10.1111/j.1365-2869.2010.00848.x

61. Takeuchi N, Uchimura N, Hashizume Y, et al (2001) Melatonin therapy for REM sleep behavior disorder. Psychiatry Clin Neurosci 55:267–269. doi: 10.1046/j.1440-1819.2001.00854.x

62. Kunz D, Bes F (1999) Melatonin as a therapy in REM sleep behavior disorder patients: an open-labeled pilot study on the possible influence of melatonin on REM-sleep regulation. Mov Disord 14:507–511.

63. Feemster JC, Smith KL, McCarter SJ, St Louis EK (2019) Trauma-Associated Sleep Disorder: A Posttraumatic Stress/REM Sleep Behavior Disorder Mash-Up? J Clin Sleep Med 15:345–349. doi: 10.5664/jcsm.7642

64. Xu Z, Oliver JR, Anderson KN (2019) Parasomnia overlap disorder with adolescent-onset presumed REM sleep behavior disorder converting to Parkinson's disease after 48 years. Sleep Med 57:97–99. doi: 10.1016/j.sleep.2019.01.041

65. Kunz D, Bes F (2017) Twenty Years After: Another Case Report of Melatonin Effects on REM Sleep Behavior Disorder, Using Serial Dopamine Transporter Imaging. Neuropsychobiology 76:100–104. doi: 10.1159/000488893

66. wierzbicka A, Szaulińska K, Psychiatry EPAI, 2017 (2017) REM behaviour sleep disorder–case study. Adv Psychiatry Neurol 26:194–202. doi: https://doi.org/10.5114/ppn.2017.70551

67. Felix S, Thobois S, Peter-Derex L (2016) Rapid eye movement sleep behaviour disorder symptomatic of a brain stem cavernoma. J Sleep Res 25:211–215. doi: 10.1111/jsr.12364

68. Anderson KN, Jamieson S, Graham AJ, Shneerson JM (2008) REM sleep behaviour disorder treated with melatonin in a patient with Alzheimer's disease. Clin Neurol Neurosurg 110:492–495. doi: 10.1016/j.clineuro.2008.01.004

69. Kunz D, Bes F (1997) Melatonin effects in a patient with severe REM sleep behavior disorder: case report and theoretical considerations. Neuropsychobiology 36:211–214. doi: 10.1159/000119383

70. Esaki Y, Kitajima T, Koike S, et al (2016) An Open-Labeled Trial of Ramelteon in Idiopathic Rapid Eye Movement Sleep Behavior Disorder. J Clin Sleep Med 12:689–693. doi: 10.5664/jcsm.5796

71. Kashihara K, Nomura T, Maeda T, et al (2016) Beneficial Effects of Ramelteon on Rapid Eye Movement Sleep Behavior Disorder Associated with Parkinson's Disease - Results of a Multicenter Open Trial. Intern Med 55:231–236. doi: 10.2169/internalmedicine.55.5464

72. Kasanuki K, Iseki E, Nishida Y, et al (2013) Effectiveness of ramelteon for treatment of visual hallucinations in dementia with Lewy bodies: a report of 4 cases. J Clin Psychopharmacol 33:581–583. doi: 10.1097/JCP.0b013e318295fdf4

73. Bonakis A, Economou N-T, Papageorgiou SG, et al (2012) Agomelatine may improve REM sleep behavior disorder symptoms. J Clin Psychopharmacol 32:732–734. doi: 10.1097/JCP.0b013e31826866f8

74. Tan A, Salgado M, Fahn S (1996) Rapid eye movement sleep behavior disorder preceding Parkinson's disease with therapeutic response to levodopa. Mov Disord 11:214–216. doi: 10.1002/mds.870110216

75. Sasai T, Inoue Y, Matsuura M (2012) Effectiveness of pramipexole, a dopamine agonist, on rapid eye movement sleep behavior disorder. Tohoku J Exp Med 226:177–181.

76. Kumru H, Iranzo A, Carrasco E, et al (2008) Lack of effects of pramipexole on REM sleep behavior disorder in Parkinson disease. Sleep 31:1418–1421.

77. Fantini ML, Gagnon J-F, Filipini D, Montplaisir J (2003) The effects of pramipexole in REM sleep behavior disorder. Neurology 61:1418–1420. doi: 10.1212/wnl.61.10.1418

78. Schmidt MH, Koshal VB, Schmidt HS (2006) Use of pramipexole in REM sleep behavior disorder: results from a case series. Sleep Med 7:418–423. doi: 10.1016/j.sleep.2006.03.018

79. Dusek P, Bušková J, Růžička E, et al (2010) Effects of ropinirole prolonged-release on sleep disturbances and daytime sleepiness in Parkinson disease. Clin Neuropharmacol 33:186–190. doi: 10.1097/WNF.0b013e3181e71166

80. Wang Y, Yang Y, Wu H, et al (2016) Effects of Rotigotine on REM Sleep Behavior Disorder in Parkinson Disease. J Clin Sleep Med 12:1403–1409. doi: 10.5664/jcsm.6200

81. Ozaki A, Nishida M, Koyama K, et al (2012) Donepezil-induced sleep spindle in a patient with dementia with Lewy bodies: a case report. Psychogeriatrics 12:255–258. doi: 10.1111/j.1479-8301.2012.00411.x

82. Brunetti V, Losurdo A, Testani E, et al (2014) Rivastigmine for refractory REM behavior disorder in mild cognitive impairment. Curr Alzheimer Res 11:267–273. doi: 10.2174/1567205011666140302195648

83. Yamamoto K, Uchimura N, Biological MHSA, 2006 (2006) Evaluation of the effects of paroxetine in the treatment of REM sleep behavior disorder. Wiley Online Library. 4:190–192. doi: 10.1111/j.1479-8425.2006.00212.x

84. Takahashi T, Mitsuya H, Murata T, et al (2008) Opposite effects of SSRIs and tandospirone in the treatment of REM sleep behavior disorder. Sleep Med 9:317–319. doi: 10.1016/j.sleep.2007.05.003

85. Bamford CR (1993) Carbamazepine in REM sleep behavior disorder. Sleep 16:33–34.

86. Onofrj M, Luciano AL, Thomas A, et al (2003) Mirtazapine induces REM sleep behavior disorder (RBD) in parkinsonism. Neurology 60:113–115. doi: 10.1212/01.wnl.0000042084.03066.c0

87. Mayer G (2016) Efficacy of sodium oxybate on REM sleep behavior disorder in a patient with narcolepsy type 1. Neurology 87:2594–2595. doi: 10.1212/WNL.0000000000003389

88. Matsui K, Sasai-Sakuma T, Ishigooka J, et al (2019) Effect of Yokukansan for the Treatment of Idiopathic Rapid Eye Movement Sleep Behavior Disorder: A Retrospective Analysis of Consecutive Patients. J Clin Sleep Med 15:1173–1178. doi: 10.5664/jcsm.7816

89. Chagas MHN, Eckeli AL, Zuardi AW, et al (2014) Cannabidiol can improve complex sleep-related behaviours associated with rapid eye movement sleep behaviour disorder in Parkinson's disease patients: a case series. J Clin Pharm Ther 39:564–566. doi: 10.1111/jcpt.12179
