# Supplementary material for: A critical review of the pharmacological treatment of REM sleep behavior disorder in adults: time for more and larger randomized placebo-controlled trials
Source: J Neurol. 2021 Jan 7;269(1):125–48. doi: 10.1007/s00415-020-10353-0 (PMC8739295; doi:10.1007/s00415-020-10353-0)
Supplement: Supplementary file 3 — (PDF 128 KB) [file 415_2020_10353_MOESM3_ESM.docx]

**Supplementary Materials:**  World Health Organisation Clinical Trial registrations on the pharmacological treatment of RBD

**Title:** An update on the pharmacological treatment of REM sleep behavior disorder in adults: Time for more and larger randomized placebo-controlled trials.

**Authors**: Moran Gilat PhD, Nathaniel Marshall PhD, Dries Testelmans MD PhD, Bertien Buyse MD PhD, Simon JG Lewis MD PhD

**Corresponding author:**

Dr Moran Gilat PhD

Email: [moran.gilat@kuleuven.be](mailto:moran.gilat@kuleuven.be)

**Supplementary Table:** World Health Organisation Clinical Trial registrations on the pharmacological treatment of RBD

| Registration ID | Registration Source | Date Registration  first posted | Study  Start date | (Estimated) Completion  Date | Prospective or Retrospective Registration | Public Title | Trial status | Published in peer-review journal? | Results  posted on registry? | Notes |
| --- | --- | --- | --- | --- | --- | --- | --- | --- | --- | --- |
| NCT01401413 | ClinicalTrials.gov | 07/2011 | 01/2008 | 09/2010 | Retrospective | Study to Determine Whether Ramelteon Helps People With REM Sleep Behavior Disorder | **Completed** | No | No | *Possible publication bias.* |
| NCT00745030 | ClinicalTrials.gov | 09/2008 | 06/2008 | 09/2010 | Retrospective | Efficacy and Tolerability of Ramelteon in Patients With Rapid Eye Movement (REM) Behavior Disorder and Parkinsonism | Terminated | No | No (only enrolment numbers) | Terminated after enrolling three subjects due to low recruitment rates. |
| 2009-012071-10 | EU Clinical Trials Registry (EUCTR) | 29/05/2009 | 15/06/2009 | Not reported | Prospective | Rivastigmine in the treatment of Sleep REM Behaviour Disorder (RBD) and Hallucinations in Parkinsonism: a Clinical and Polysomnographic study | **Completed** | *Possibly* | N.A. | Limited information on trial registration. |
| ACTRN12613000648729 | Australian New Zealand Clinical Trials Registry (ANZCTR) | 02/06/2013 | 01/08/2013 | 15/01/2018 | Prospective | Efficacy of melatonin in rapid eye movement (REM sleep) behaviour disorder with coexisting Parkinsonism | **Completed** | Yes | N.A. |  |
| ACTRN12613000647730 | Australian New Zealand Clinical Trials Registry (ANZCTR) | 02/06/2013 | 01/08/2013 | 15/05/2016 | Prospective | Efficacy of melatonin in idiopathic REM sleep behaviour disorder | Terminated | No | No | Terminated after enrolling 6 subjects due to low recruitment rates. |
| NCT02312908 | ClinicalTrials.gov | 09/12/2014 | 01/03/2015 | 01/03/2016 | Prospective | Effect of Clonazepam on REM Sleep Behavior Disorder in Patients With Parkinsonism | **Completed** | Yes | N.A. |  |
| NCT02708186 | ClinicalTrials.gov | 03/2016 | 03/2016 | 05/2018 | Prospective | Study Evaluating Nelotanserin for Treatment of REM Sleep Behavior Disorder in Subjects With Dementia (DLB or PDD) | **Completed** | No | Partly | *Possible publication bias.* |
| NCT02789592 | ClinicalTrials.gov | 06/2016 | 07/2016 | 12/2019 | Retrospective | Efficacy and Safety of Melatonin PR and Clonazepam in Patients With REM Sleep Behavior Disorder in Parkinson Disease | **Unknown** | No | No | *Possible publication bias.* |
| NCT02836743 | ClinicalTrials.gov | 19/07/2016 | 01/2016 | 31/12/2017 | Retrospective | Effect of Slow-release Melatonin (Circadin®) Therapy on Idiopathic RBD: a Pilot Study | **Completed** | Yes | N.A. |  |
| NCT02871427 | ClinicalTrials.gov | 18/08/2016 | 20/10/2016 | 17/01/2019 | Prospective | Open-label Study of Nelotanserin in Lewy Body Dementia With Visual Hallucinations or REM Sleep Behavior Disorder | Terminated | No | No | Terminated due to changes to study drug development program. |
| NCT03255642 | ClinicalTrials.gov | 21/08/2017 | 09/11/2017 | 30/12/2019 | Prospective | Efficacy and Safety of Melatonin and Clonazepam for IRBD | Recruiting | N.A. | N.A. | Trial ongoing past expected completion date. |
| RBR-5fwhf7 | Brazilian Registry of Clinical trials (REBEC) | 22/11/2017 | 01/08/2017 | 01/01/2018 | Retrospective | Canabidiol in the treatment of sleep disorder associated with Parkinson's Disease | Recruiting | N.A. | N.A. | Trial ongoing past expected completion date. |
| ChiCTR1800017395 | Chinese Clinical Trial Registry (ChiCTR) | 28/07/2018 | 01/09/2018 | 31/12/2020 | Prospective | Clinical research about neuroprotection of idebenone on rapid eye movement sleep behaviour disorder | Recruiting | N.A. | N.A. | Trial ongoing. |
| UMIN000034492 | Japan Primary Registries Network (JPRN) | 15/10/2018 | 15/10/2018 (anticipated) | Not reported | Prospective | The effect of Orexin receptor antagonist Suvorexant on REM sleep behaviour disorder using Escitalopram (2) | Not yet Recruiting | N.A. | N.A. | Trial not yet recruiting as per 10/2018. |
| NCT04006925 | ClinicalTrials.gov | 05/07/2019 | 10/10/2019 | 01/12/2020 | Prospective | Treatment of REM Sleep Behavior Disorder (RBD) With Sodium Oxybate | Recruiting | N.A. | N.A. | Trial ongoing. |
| KCT0004317 | Clinical Research Information Service (CRIS) of Korea | 25/09/2019 | 02/03/2020 (anticipated) | 31/12/2025 | Prospective | Effects of Donepezil or Rivastigmine in Patients with idiopathic REM Sleep Behavior Disorder Patients | Not yet recruiting | N.A. | N.A. | Trial ongoing. |
| IRCT20170821035819N3 | Iranian Registry of Clinical Trials (IRCT) | 16/02/2020 | 22/06/2019 | 20/02/2020 | Retrospective | Comparison of the effect of Melatonin and Clonazepam on the REM sleep behavior disorder of patients with Parkinson's disease | **Completed** | No | No | Investigators may be in the process of publishing the findings. RBD listed as a secondary outcome. |

***NOTES: The search was conducted on the International Clinical Trials Registry Platform of the World Health Organisation (apps.who.int) on 20 November 2020 using (“REM sleep behavior disorder” OR “RBD”) as search terms. The search resulted in 77 listed trials, of which 23 potentially eligible trials were selected based on title screening. Following full registration screening, a total of 17 trials were deemed eligible for assessment of publication bias and added to this Table. Three trials from Japan (JPRN-UMIN0000/20313/27333/37876) were excluded as the participants only took the investigational drug on a single night, and three trials from China (NCTR0/4152655/4534023 and ChiCTR2000037624) were excluded for not targeting symptomatic RBD.***
